# Observing Change Over Time in Strength-Based Parenting and Subjective Wellbeing for Pre-teens and Teens

**DOI:** 10.3389/fpsyg.2019.02273

**Published:** 2019-10-10

**Authors:** Lea Waters, Daniel J. Loton, Dawson Grace, Rowan Jacques-Hamilton, Michael J. Zyphur

**Affiliations:** ^1^Centre for Positive Psychology, The University of Melbourne, Melbourne, VIC, Australia; ^2^Department of Management and Marketing, The University of Melbourne, Melbourne, VIC, Australia

**Keywords:** wellbeing, parenting, strengths, adolescence, pre-teens, teens, positive psychology

## Abstract

The focus of this study was on adolescent mental health. More specifically, the relationship between strength-based parenting (SBP) and subjective wellbeing (SWB) during adolescence was examined at three time points over 14 months (*N* = 202, *M*_age_ = 12.97, *SD*_age_ = 0.91, 48% female). SBP was positively related to life satisfaction and positive affect at each of the three time points, and was negatively related to negative affect. SBP and SWB both declined significantly over time. When examining the causal relationships between SBP and SWB, two different statistical models were applied: latent growth-curve models (LGM) and random-intercept cross-lagged panel models (RI-CLPM). The LGM revealed a strong positive relationship between changes in SBP and SWB. Specifically, this model showed that SBP at one time point predicted adolescent SWB at future time points. However, when the more stringent statistical test was completed through RI-CLPMs, no cross-lagged paths reached significance. Thus, while parenting is a significant predictor of wellbeing for pre-teens and teens in real time, it is not predictive of wellbeing at future time points. Parents, thus, cannot assume that their current levels of SBP are ‘banked’ by their children to support future wellbeing. Instead, SBP needs to be an ongoing, contemporary parenting practice. Furthermore, the fact that perceptions of SBP decline in this age bracket suggest that SBP interventions may be helpful in supporting adolescent mental health.

## Introduction

“*A healthy family is necessary for a healthy society*”

(Shapiro, 2004, p. 27)

The teen, and now increasingly pre-teen, years are characterized by intense changes to a young person’s physical development, identity, social life, family relationships, exposure to drugs and alcohol, academic requirements, employment and economic responsibilities (Larson et al., 1996; Singh, 1998; Levy-Warren, 1999; Brown, 2004; Sisk and Foster, 2004; Viner and Taylor, 2007; Andersen and Teicher, 2008; Hussain et al., 2008; Sodha and Margo, 2008; Lerner and Steinberg, 2009; Sodha, 2009). As such, the second decade of life sees a young person needing to build up the psychological, emotional, and social capacities necessary to meet the demands of these changes and grow into adulthood. While these skills and dispositions develop continuously from childhood, adolescence has been recognized as a particularly sensitive developmental period for these skills to be gained (Pfeifer et al., 2011; Somerville, 2013) given the shift to more independence (Feldman and Elliott, 1990), greater life complexity (Crone and Dahl, 2012), the teen brains heightened neuroplasticity (Andersen and Teicher, 2008; Giedd, 2008), and the fact that this is the life phase immediately prior to adulthood/emerging adulthood (Steinberg, 2014). Indeed, Patton et al. (2016) state that adolescence is “a critical phase in life for achieving human potential” (p. 2324) as it is the life stage “in which an individual establishes the social, cultural, emotional, educational, and economic resources to maintain their health and wellbeing across the life course.” (p. 2427).

Parents are critical in shaping an adolescent’s health and wellbeing (Bruyn et al., 2003; Galambos et al., 2003; National Research Council, 2011; Bøe et al., 2018). Yet, in their review of adolescent health and wellbeing, Patton and his colleagues assert: *“*given that families and parents remain the most important figures in the lives of most adolescents, the paucity of rigorous research into family influences on adolescent health and wellbeing is a striking knowledge gap” (Patton et al., 2016, p. 2432). Adding to the critique of Patton et al., parenting research has been criticized for not adequately studying how parent–child dynamics change over time (Holden and Miller, 1999; Rimehaug et al., 2011) and for having a deficit focus that has concentrated on effects of negative parenting factors (e.g., parent addiction, violence, mental illness, or neglect) at the expense of understanding the effects of positive parenting factors (e.g., compassion, strengths, emotional atonement) (Sheridan and Burt, 2009; Conoley et al., 2015; Waters and Sun, 2016).

The current study focuses on mental health outcomes for adolescents and sits within the paradigm of positive psychology to explore the dynamic effect of strength-based parenting (SBP) on subjective wellbeing (SWB) in pre-teens and early-to-mid teens across a 3-wave longitudinal study.

### Youth Wellbeing

Research shows that the greatest risk for developing mental illness occurs in the second decade of life (Costello et al., 2003; Slade et al., 2009; Kieling et al., 2011; De Girolamo et al., 2012; Schwartz et al., 2012; Eyre and Thapar, 2014; Patton et al., 2014). Costello et al. (2011) found that there is an increase in rates of panic disorder, agoraphobia, substance use disorders and depression from childhood to adolescence.^1^ Kessler et al. (2001) epidemiological research revealed a rise from 1% depression in the population under age 12 to 17–25% of the population by the end of adolescence. In early adolescence, epidemiological research shows that mental disorders sit around 10% (10.9%, Anselmi et al., 2010; 9.8%, Frigerio et al., 2009) but rise to 13.4–21.8% in mid to late adolescence (Costello et al., 2011; Polanczyk et al., 2015).

Epidemiological data from Costello et al. (2003, 2011) revealed the most common diagnoses in adolescents to be substance abuse disorders (12.1%), anxiety disorders (10.7%), depressive disorders (6.1%) and behavioral/spectrum disorders (e.g., conduct disorder, oppositional defiant disorder, attention-deficit hyperactivity disorder; 3–4%). The World Health Organization (2014) lists depression as the number one cause of illness in adolescence and according to Andersen and Teicher (2008) “depression emerges with force and frequency in adolescence” (p.183). Tragically, suicide is a leading cause of death among teenagers worldwide (Hawton et al., 2012).

Naturally, the question has arisen as to whether the prevalence of mental illness has increased over the decades. The evidence is mixed, and while some researchers have found no evidence for change (Costello et al., 2006), the majority of studies find mental illness has increased (Rutter and Smith, 1995; Collishaw et al., 2004, 2010). For example, in the United States of America (USA), Twenge’s research has been convincing in showing that mental illness in adolescents and early adults has increased over the generations. For example, Twenge et al. (2010) analyzed generational comparisons using the Minnesota Multiphasic Personality Inventory (MMPI) across college students from 1938 to 2007 (*N* = 63,706) and across high school students from 1951 to 2002 (*N* = 13,870) and found that fives time as many of the youth from the later cohorts scored above common cut-offs for psychopathology than compared to the earlier generations.

In another study of generational comparisons across high school and college cohorts from a 1980–1990 cohort to a 2000–2010 cohort (*N* = 6.9 million), Twenge et al. (2015) found that the 2010 cohort were twice as likely to report symptoms of mental illness than teens in 1980s. The more recent cohorts also reported greater trouble sleeping, more feelings of being overwhelmed and were twice as likely to have seen a professional for mental health issues. College students from the later decades were more likely to report feeling overwhelmed and to believe they were below average in mental health compared to earlier generations of college students. The suicide rate dropped from 1991 to 2011 but the researchers suggested “subtle symptoms of depression became more prevalent even as some overt indicators of depression became less prevalent” (p. 437).

Finally, Twenge et al. (2018) used two national data sets from 2010 to 2015 to study if there were any shifts in depression, suicidal-outcomes (e.g., making a plan, attempting suicide) and suicide for American adolescents (*N* = 506,820). The researchers found that depression increased by 33%, suicide-related outcomes rose by 15% and suicide rose by 31%. These increases in mental health issues were consistent across race/ethnicity, SES, region, and age/grade. However, there were gender effects with females showing greater increases than males.

These trends in the USA are similar those in the United Kingdom (UK) as shown by Collishaw and his colleagues who have conducted a number of independent cross-cohort comparisons of adolescent mental illness in the UK. In one study, Collishaw et al. (2004) compared levels of depressed mood, anxiety and fearfulness (as assessed by parents) in 15/16-year olds and found a substantial increase from 1986 to 1999. Indeed, the proportion of parents reporting mental health symptoms increased by 70% between 1986 and 1999. In a later study, Collishaw et al. (2010) found increases in symptoms of anxiety and depression from 1986 to 2006 based on youth self-report with twice as many adolescents reporting five or more symptoms of anxiety or depression in 2006 compared to 1986 (15% vs. 7%). Other UK researchers report similar findings and Sweeting et al. (2009) identified that the number of young people meeting established GHQ ‘case criteria’ almost doubled for Scottish boys and more than doubled for Scottish girls between 1987 and 2006.

In 2015, Collishaw broadened his research sample beyond the UK to look at international trends arising in UK, Finland, the Netherlands and other Nordic countries from 1977–2011. Data across 21 studies revealed that clinical diagnosis and treatment of adolescent psychiatric disorders, problems and antisocial behavior increased in recent decades. Another international study conducted by Bor et al. (2014) examined long-term time trends (>10 years) in mental health problems for adolescents across seven countries (Finland, Iceland, Sweden, Scotland, UK, USA, and China) and concluded that “The burden of externalizing problems appears to be stable… However, the findings for internalizing problems suggest an increasing symptom burden in recent cohorts of adolescents, especially girls” (p.614).

### Strengths and Youth Wellbeing

It is evident from the data above, that efforts are needed to assist adolescents to build up their wellbeing. One factor that has been shown to be significantly, positively related to wellbeing in young people is that of strengths (Proctor et al., 2011; Suldo et al., 2014). Govindji and Linley (2007) define strengths as “the things you are able to do well or do best” (p. 146). Research into strengths builds upon earlier humanistic psychological research into personality, abilities, self-actualization, virtues and character (Kristjánsson, 2012).

Strengths are one’s capacities for excellence and talent, together with one’s positive personality traits that play out in persistent patterns of person-environment interaction including one’s thoughts, actions and activities (Peterson and Seligman, 2004; Biswas-Diener et al., 2017; Waters, 2017). Strengths provide a person with a sense of energy and efficacy and are used to support one’s goals, values and growth (Peterson and Seligman, 2004; Linley et al., 2010).

The scientific study of strengths is a growing body of research (Donaldson et al., 2015) and large-scale bibliometric analyses of the positive psychological literature by Rusk and Waters (2015) demonstrated that the study of strengths has grown considerably in scientific interest since 1992. Indeed, it is the highest growing topic within the field (other rising topics include life satisfaction, positive affect, self-determination and optimism) (Rusk and Waters, 2015).

To date there have been two broad approaches used in the study of strengths. The first approach, a content approach, focuses the types of strengths that people have. This approach has spawned the development of various strengths frameworks and assessments outlining a range of different strengths be they moral qualities (e.g., kindness, courage) (Peterson and Seligman, 2004; see the Values in Action Survey^2^), natural talents combined with knowledge and skills (e.g., maximizer, adaptable; Rath, 2007; see StrengthsFinder^3^) or qualities that are energizing and allow for optimal functioning (e.g., authenticity, narrator; Linley et al., 2010; see Realise2^4^).

The second approach, the process approach, moves away from categorizing types of strengths and instead considers the underlying processes that are used to develop strengths. Govindji and Linley (2007), for example, focus their research on developing strengths through two key processes: (1) strength knowledge which they define as a person’s “awareness and recognition of their strengths” (p. 146), and (2); and strengths use, which is defined as the extent to which individuals “use their strengths in a variety of settings” (p. 147). Recently, Biswas-Diener et al. (2017) called for a third element beyond knowledge and use to be included in the process-approach to strengths, that of strengths development.

In adult samples (Park, 2004) and youth samples (Park and Peterson, 2006a), knowledge and use of certain character strengths (hope, love, gratitude, and zest) have been associated with greater life satisfaction and, furthermore, the same strengths in parents predict greater LS in their children (Park and Peterson, 2006b). In a meta-analysis of 14 articles (29 effect sizes) on character strength interventions in adult and adolescent samples, Schutte and Malouff (2018) identified significant relationships between knowing and using one’s strengths with life satisfaction (weighted Hedges’ g of 0.42) and positive affect (weighted Hedges’ g of 0.32).

Youth samples show significant relationships between strengths and SWB. For example, in the United States, Suldo et al. (2014) studied the impact of a 10-week school-based wellness-intervention embedded with strengths in pre-teens (aged 10–12) and found significant improvements in life satisfaction after the program. In a sample of 12–14 year-olds in the UK, Proctor et al. (2011) found increases in life satisfaction following a 24-lessons ‘Strengths Gym’ program. Moving up to older teens and early adults, a study in China found that college students who undertook an 18-week elective course on strengths showed statistically higher levels of life satisfaction after the course than before, and compared to those who did not complete the course (Duan et al., 2014).

The meta-analysis and youth studies cited above show that strengths interventions increase SWB and life satisfaction and, thus, offer a promising route to mental health for teenagers. However, not every young person has the good fortune to participate in a strengths-intervention at school or college and, thus, other more naturally occurring opportunities that build strengths in young people need to be explored.

To this end, research shows that one powerful way to build strengths in a young person is through the strengths feedback they receive from others in their everyday life. For example, Spreitzer et al. (2009) found that when teenagers receive strengths feedback from teachers, coaches, bosses, friends and family it boosts their wellbeing. Importantly, this research showed that strengths feedback from both professional (teachers, coaches, bosses) *and* personal sources (family and friends) was more important than strengths feedback from professional sources alone, suggesting that parents play an important role in teens learning about their strengths.

The finding that other people can be even more accurate than the self at predicting certain trait-relevant behaviors and abilities (Vazire and Mehl, 2008; Vazire, 2010) suggests that others sometimes know us better than we know ourselves. In particular, few experiences in life rival the extensive insight gained about another human being than that of a parent raising their child. As parents have a myriad of daily opportunities to notice which situations and activities their child enjoys, is energized by and performs well in, they are uniquely placed to provide feedback to their teenager about his or her strengths.

### Parenting and Youth Wellbeing: Strength-Based Parenting

Parenting has been a topic of empirical psychological inquiry since the 1960’s and over the past 50+ years researchers have examined a range of ways in which parenting affects a child’s mental health, adjustment, brain development, and trajectory into adulthood (for reviews see Steinberg, 2001; Mejia et al., 2012).

Historically, the research has focused on the effects of harmful parenting with studies investigating the impact of parental control, punishment, coldness, neglect, violence, conflict, addiction and mental illness on child and teen outcomes (Steinberg, 1987; Erel and Burman, 1995; Lovejoy et al., 2000; Caprara et al., 2002; Barnard and McKeganey, 2004; Dubowitz and Bennet, 2007; Ma et al., 2012). The impact of such parenting has been shown to lead to increased risk of psychopathology, suicidal ideation, substance abuse, delinquency, aggression, externalizing disorders, teen pregnancy and criminal behavior as well as deficits in social and emotional functioning (Kinard and Klerman, 1980; Baumrind, 1991a; McCloskey et al., 1995; Kaplan et al., 1999; Mejia et al., 2012). Beyond the focus on negative parental states (e.g., addiction, mental illness) and negative parenting practices (e.g., punishment, discipline, violence, neglect), environmental adversities and family level adversities such as poverty, poor education levels of parents and family stress have also been shown to be related to negative outcomes for adolescents including peer problems, emotional and conduct problems, inattention, mental health problems and psychiatric disorder (Bøe et al., 2012, 2018).

Prospective and retrospective studies have shown that negative parenting during adolescence leads to a raft of harmful outcomes not only in one’s youth but also through into adult life including poorer adjustment to college, marriage, and to becoming parents themselves, as well as greater risk of heart attack, alcoholism, and obesity (Holmes and Robins, 1988; Mullen et al., 1999; Vaillant and Mukamal, 2001; Willinger et al., 2005; Schnuck and Handal, 2011; Bentley and Widom, 2012). Sadly, longitudinal research shows that adults with a history of parental maltreatment in their childhood are three times more likely to have depression and suicidality (Brown et al., 1999).

The research above has motivated an early intervention approach through the design of parenting programs used with at-risk families. One such example is the Family Strengthening Program, a strength-based program for families in crisis and for whom child safety concerns have been identified. This program involves therapists working with parents to teach them solution-focused strategies and unearth the assets within the family that can be used to create safer, healthier patterns. The Family Strengthening Program been shown to improve family safety, family health, family interaction, and child wellbeing (Katsikitis et al., 2013).

In addition to working with families at risk of harm, programs have also been designed to assist parents who may have high levels of parental stressors due to raising children who have challenges such as autism, developmental disabilities, intellectual disabilities, anxiety disorders and conduct disorders (Feldman, 1994; McConachie and Diggle, 2007; Plant and Sanders, 2007; Cartwright-Hatton et al., 2011). The Positive Parenting Program (Triple P) is one successful example that has been utilized in families with children who have behavioral, emotional, and/or developmental problems (Sanders et al., 2000). This program teaches parents how to praise pro-social behavior, minimize coercion and reduce opportunities for problem behavior (e.g., by providing ground rules). The meta-analysis of de Graaf et al. (2008) found the Triple P parenting program successfully reduces disruptive behaviors in children.

Other strength-based movements in family therapy include ‘family centered services’ (Dunst and Deal, 1994) and ‘resilient families’ (McCubbin et al., 1999; Shortt et al., 2007) both of which employ positive processes to reduce negative outcomes for families undergoing adversity and/or crisis.

Looking at the above research, it is no surprise to learn that the parenting literature has been criticized for its bias toward studying negative factors in terms of the type of families studied (families in distress/dysfunction/crisis; families at-risk, low SES families) and in terms the outcomes investigated (e.g., mental illness, substance abuse, conduct disorders, behavioral disorders). The programs used, while positively oriented, are designed to focus upon family problems (e.g., behavioral disorders) and are measured in terms of the ability to reduce *negative outcomes* (e.g., conduct disorders). Indeed, according to Shapiro (2004), much of the existing research in parenting is characterized by “one-sided and negative views of family process” (p. 33). In line with this, Sheridan and Burt (2009) assert that “Most research in children and families maintains a deficit focus; it concentrates more on the role of risk factors than assets.” (p.552). Similarly, Katsikitis et al. (2014) argue that “Much of the focus of mother-daughter relationships in the literature has been on strategies to manage and deal with negative adolescent behavior” (p. 1).

While a great deal is now known about the adverse impacts of being raised with parental abuse, neglect, mental illness, conflict and the like; and while we have learnt about the impact that positive parenting approaches can have on *ameliorating negative outcomes*, such as harm in at-risk families and conduct issues in certain sub-samples of children, surprisingly little is known about how to support parenting approaches that create positive outcomes for mainstream families who are not at risk and are not dealing with adversities such as poverty or children with challenging behaviors. Yet, when considering Seligman’s (1999) call, that psychology should be able to help document the factors that promote flourishing families (not just support struggling families), it is time for parenting research to study the ways in which we can enhance positive outcomes in everyday families. Given that mainstream families (i.e., families who do not meet the clinical levels for dysfunction or pathology) represent the biggest proportion of families in society, Seligman’s call motivates us to apply parenting research to the whole community and not just those with difficulties.

One pioneer in the application of positive parenting in non-clinical families is Steinberg who, together with his colleagues, has been studying the effect of autonomy-granting parenting since the 1970’s in everyday families across multiple cultures^5^ (Steinberg et al., 1992; Gray and Steinberg, 1999; Steinberg, 2001; Steinberg and Morris, 2001; Steinberg, 2014). Autonomy-granting parenting refers to the extent to which parents allow teens to develop their own opinions and beliefs and is characterized by three parental elements: warmth, boundaries/firmness, and autonomy granting (Steinberg, 2000). Autonomy-granting parenting has been related a host of beneficial outcomes for children and teens including higher self-esteem, social-confidence, subjective wellbeing, self-reliance, achievement motivation and school grades (for a review see Steinberg, 2001). Steinberg’s work has long shown us the benefits of positive parenting.

Other positively oriented research can be found in the USA with Baumrind’s work on authoritative parenting, defined as warm and firm (Baumrind, 1991b) and the research by Conger et al. (1992) who showed that positive behavior in mothers (e.g., nurturing and involvement) predicted school performance, self-confidence and peer relationships. In Australia, Havighurst and her colleagues have found that when parents are taught how to emotionally tune into their children they are more skilled at discussing causes and consequences of emotions and this leads to fewer internalizing issues with their children (Havighurst et al., 2010; Kehoe et al., 2013). In another Australian study, a longitudinal prospective study that tested the relationship between parent communication and brain development showed that positive parent communication (defined as a pattern of communication where the parent is approving, validating, affectionate, humorous, happy, pleasant, and caring) was associated with beneficial brain growth that enhances capacity for learning, decision-making, social skills, and emotional functioning (Whittle et al., 2014). As part of the Bergen Child Study in Norway, Bøe et al. (2014) found that affirmative parenting practices such as love, affection, praise, rewarding and respect as well as giving help and support when the adolescent is stressed (Last et al., 2012) were inversely related to externalizing problems, such as conduct problems and hyperactivity-inattention, together with internalizing problems, such as peer problems and emotional problems, in 11–13 years olds. Moreover, these positive parenting practices together with the parent’s own emotional wellbeing, mediated the relationship between the family’s socio-economic status and the degree to which the early adolescents were reporting symptoms of externalizing and internalizing disorders. Finally, a two-wave retrospective study in the UK on well-being in midlife women showed that the effects of positive parenting from childhood persisted into adulthood, with higher levels of parental care being associated with higher psychological well-being in mid-life (Huppert et al., 2010).

Although the last five decades have tipped more to the deficit end of parenting research, there have been pockets of positively oriented research as identified above. More recently, the advent of positive psychology, through its umbrella effect of gathering together positively oriented science into an aligned movement (Rusk and Waters, 2013, 2015) has provided the impetus and platform for a larger group of researchers to study factors that create thriving families (Shapiro, 2004; Sheridan et al., 2004; Katsikitis et al., 2013; Waters, 2017). Indeed, positive psychology, with its appreciative outlook on human virtue, resilience and potential, provides parenting researchers with a broad canvas upon which to explore the positive and pro-social side of family life. Several new strands of positive psychology research are being applied in parenting and family research both in clinical/at risk families and mainstream families. These strands of research include mindful parenting (Dumas, 2005; Geurtzen et al., 2015; Waters, 2016), family centered positive psychology (Sheridan et al., 2004; Sheridan and Burt, 2009), positive family therapy (Shapiro, 2004; Conoley et al., 2015), empathy (Farrant et al., 2011), compassion-focused parenting (Neff and McGehee, 2010; Kirby, 2017; Kirby and Baldwin, 2018) and SBP (Waters, 2015a, b, 2017; Waters and Sun, 2016; Jach et al., 2017; Loton and Waters, 2017; Waters et al., 2019).

Some of these strands of inquiry are still in the conceptual phase (e.g., family centered positive psychology, positive family therapy) and most are in the early stages of empirical research, which is to be expected given that positive psychology is still a relatively new field. At this stage in time, SBP has the highest number of peer reviewed empirical publications of the positive psychology parenting topics outlined above and is the focus of the current three-wave field study.

Waters (2015a) defined SBP as an approach to parenting that “deliberately identifies and cultivate positive states, positive processes and positive qualities in children” (p. 690). Rather than take a content approach to strengths, SBP follows the process approach in that it focuses on how parents can help their children develop and improve their strengths, regardless of what the type of strength is (Waters, 2017). The ‘cultivate’ element of the SBP definition includes both the ‘use and develop’ aspects of a process model of strengths and, thus aligns with both Govindji and Linley’s (2007) process model (knowledge and use) as well as the model of Biswas-Diener et al. (2017; identify-use-development). SBP has been found to be a distinct parenting construct to autonomy-granting parenting (Waters, 2015b; Loton and Waters, 2017).

The effects of SBP on children, teenagers and parents have been examined using a range of different methods including survey research, vignette studies, dyadic studies, intervention studies, longitudinal studies, and panel designs in sample sizes ranging from 100 to over 11,300 (Waters, 2015a, b; Jach et al., 2017; Loton and Waters, 2017; Sağkal and Özdemir, 2019). Research on SBP has identified two overarching findings: (1) SBP is a *protective factor* that is inversely related to anxiety, depression, stress, and negative emotions; and (2) SBP is an *enhancing factor* that is positively related to life satisfaction, self-confidence, subjective wellbeing, positive emotions, and academic grades. The relationship between SBP and youth mental health is mediated by engagement, self-efficacy, persistence and mental toughness (Loton and Waters, 2017; Sağkal and Özdemir, 2019; Waters et al., 2019). Mindset has been found moderate the relationship between SBP and strength use in teens (Jach et al., 2017). Research in the effects of SBP on the mental health of parents shows that SBP boosts parental self-efficacy and positive emotions in the parents (Waters and Sun, 2016). Research in the effects of SBP on the family level happiness shows that SBP interventions raise happiness in families (Waters, in press).

### Parental Stability Over Time

Given the significant influence a parent has on their pre-teen and teen’s wellbeing, the question of the degree to which a parent displays consistent parenting over time is an important one.

Currently, there are two competing viewpoints held as to the temporal stability of parenting. The invariant viewpoint has historically been the most dominant in the literature and holds that parenting is trait-like and, thus, stable over time. As an advocate of the invariant approach, Maccoby (1984) argued that “We can assume that the family system, like any system, has self-stabilizing properties [horizontal ellipsis]. Families stabilize around habitual patterns of interaction; thus, there is continuity over time in the familial forces” (p. 326). The assumption of temporal consistency in parenting allows researchers to assume that child-rearing approaches assessed at one point in time are a stable reflection of future parenting and can, thus, be used to predict child outcomes over time (e.g., Baumrind, 1991a; Maccoby, 1992; Steinberg et al., 1994; Holden, 1997).

An alternate school of thought characterizes parenting as consistent *and* mutable, arguing that parental behavior is influenced not only by parent traits but also by the changes in needs and behaviors of the child over time^6^ (Bornstein et al., 2008). While recognizing that a certain proportion of parenting is constant over time, this approach also explores the adjustments and changes that parents make in their approaches as their children grow. To this end, Madigan et al. (2016) assert that “It is necessary for the field to move beyond an exclusive focus on stability” (p. 122).

Holden and Miller (1999) conducted a meta-analysis on studies that had assessed parenting invariance-stability in infancy and childhood (babies: 0 months to 11 months/infants: 1–2 years/children: 3–11)^7^, and found that maternal behavior was moderately enduring over time (*r* = 0.45) but was also “a moving picture of parent-child interaction” (p. 243). In other words, parenting is both stable *and* changeable. In another test of variance-invariance in maternal patterns during in infancy and early childhood, Madigan et al. (2016) conducted a three-wave study over 36 months and concluded that stability and variability co-exist in parenting during the young years.

When it comes to the pre-teen and early-teen years, research shows the same pattern of variance-invariance and a notable trend toward more negative parenting styles as compared to earlier childhood years (Collins, 1990; Rimehaug et al., 2011). A particular pattern identified in the research is that the decline in parenting is most prominent during early adolescence where control, conflict, power, prohibition, and secrecy rise, while support, knowledge about the child, and closeness decrease (Stattin and Klackenberg, 1992; McGue et al., 2005; De Goede et al., 2009; Keijsers et al., 2010; Keijsers and Poulin, 2013). According to Levy-Warren (1999), this dip in early adolescence is likely to be due to the teen’s drive for autonomy and individuation which leads to increases in conflict and parent–child distance (Levy-Warren, 1999).

The pattern of negative changes in the parent-teen relationship alters as the teen becomes older and the parent-child relationship has been re-negotiated to one of power symmetry, where research shows closeness, respect, and trust between teen and parent rises while conflict and control diminish in the last teen years (Smollar and Youniss, 1985; Steinberg, 2001; De Goede et al., 2009).

A quick glance over the parenting variables examined above such as control, prohibition, and power, reveal a prevalence of negatively oriented constructs being tested in temporal teen-parent studies. But what of the stability of more positively oriented constructs of parenting? Does positive parenting stand the test of time or does it also take a ‘nose dive’ in the teenage years? Here, it is fair to say, the answer is still unclear as there have been few studies exploring the changes to positive aspects of parenting over time. The small amount of research that has been done, however, suggests that positive aspects of parenting decline during the pre-teen and early teen years. This has been found for parental warmth (McGue et al., 2005; Rodríguez et al., 2014^8^; Walkner and Rueter, 2014), parental closeness (Walkner and Rueter, 2014), parental support (Hafen and Laursen, 2009), as well as parental involvement, closeness and regard (McGue et al., 2005) – all of which decrease in pre-teen and teen years.

No studies have yet examined naturalistic change in SBP across time. While SBP has been treated as a trait-like construct in past studies (Waters, 2015b; Waters et al., 2019) it is also possible that SBP, like many other aspects of parenting, changes across the pre-teen and teen years. The reduced knowledge of parents about their teens, together with reduced teen-parent closeness may provide parents with fewer opportunities to see and acknowledge their teen’s strengths. Increased conflict between parents and their teens may make it more challenging for parents to see the strengths in their child, and/or may make the teen feel the conflict is occurring because the parents is only seeing their problem behaviors and is not acknowledging their positive qualities. This could result in SBP declining in the pre-teen and early-to-mid teen years. Increased self-doubt in teenagers may also mean that, even if the parent remains constant in their strength-based approach, the teen is not able to consistently absorb and integrate the positive feedback. For these reasons, and in-line with the research on parenting invariance during the pre-teen and teen years, especially the finding that other positive aspects of parenting decline, it is reasonable to assume that SBP will decline through the pre-teen and into the early teen years.

### The Current Study

Prior studies of SBP have relied on single-time-point or two-time-point designs (Waters and Sun, 2016; Sağkal and Özdemir, 2019; Waters et al., 2019). While these studies have usefully identified measurement characteristics, mediating and moderating factors, and wellbeing outcomes of SBP, more research is required to test if SBP naturalistically changes over time and, additionally, how the relationship between SBP and wellbeing changes over time. One means by which researchers can empirically investigate the dynamic causal relationship between SBP and SWB is by observing factors over multiple time points and establishing temporal precedence (McArdle, 2009; Hamaker et al., 2015). That is, whether a change in one variable tends to precede a change in another, which supports separable causal directionality and is one criterion for understanding causality. This study primarily aims to extend research on SBP by examining change in SBP and SWB at three time points over a period of 14 months in a sample of pre-teens and teens. The study is guided by four hypotheses.

Hypothesis One: Consistent with prior studies showing reductions in life satisfaction in teen samples, adolescent SWB is predicted to show a decline over time.
Hypothesis Two: Consistent with past research showing a reduction in positive parenting during the teen years, it is hypothesized that SBP will decline over time.
Hypothesis Three: Over time, adolescents who experience decreases in SBP will also tend to experience decreases in SWB, meaning these factors are likely to change together.
Hypothesis Four: After accounting for within-person stability and auto-regressive effects, changes in SBP will cause concomitant changes in SWB in the form of past SBP predicting future SWB.

## Materials and Methods

### Sample

The sample for this study comprised students across years 7–9 from a public secondary school in Victoria, Australia. The sample formed part of a three-wave study and two previously published cross-sectional studies have been published from this data set (Jach et al., 2017; Waters et al., 2019). The current paper investigates the causal and longitudinal effect of SBP on SWB, in the sample of students who successfully completed all three waves of the survey.

The school from which the sample was drawn has a socio-economic index equal to the Australian average, indicating it is representative in terms of socio-educational advantage. Two hundred and two adolescents between the ages of 12–15 completed all three waves of data collection and formed the current sample (*M*_age_ = 12.97, *SD*_age_ = 0.91, 48% female, 49.5% male, 2% preferred not to say and 1 selected ‘other’ gender). Unequal numbers of participants participated across the year levels (*N* = 113 or 55.9% Year 7s, *N* = 39 or 19.3% Year 8s, and *N* = 50 or 24.8% Year 9s; χ^2^ = 47.36, *p* < 0.001). It is unclear why response rates were lower in years 8 and 9 but it could be due to timetabling issues when the survey was completed in class time. Potential sample bias was analyzed (see Sample Bias Due to Attrition) below with tests comparing participants who completed all three waves, to those only completing one or two. Of the four variables across three points in time, only one variable (PA at time two) was significantly different between responders and non-responders indicating that response bias was not an issue in this sample.

### Procedure

Students completed a 30-min online survey during school hours across the three time points and their surveys were matched based on a unique ID number. Applying listwise deletion, 15 cases were removed due to missing data. Data collection took place in May, 2016, November, 2016, and July 2017. The average number of days elapsed between data collection waves from baseline to wave 2 was *M_days_* = 184.77 (*SD_days_* = 9.89) and number of days between wave 2 to wave 3 was *M_days_* = 241.1 (*SD_days_* = 3.66). The average total time elapsed from baseline to the third wave of measurement was (*M_days_* = 426.49, *SD_days_* = 8.46, *M_months_* = 13.50, *SD_months_* = 0.50).

All procedures in this study complied with the National Statement on Ethical Conduct in Human Research and were approved by the University of Melbourne’s Human Research Ethics Committee, and school Principal. Standing informed consent was provided by parents, through a process of advertising the study in the school newsletter and website prior to data collection, with an option to for their child opt-out. Participating students were also given active ‘assent’ to withdraw from the study and were advised by the classroom teacher and school Wellbeing Coordinator immediately preceding in-class data collection that the study was voluntary.

### Measures

#### Strength-Based Parenting Knowledge

The 7-item SBP-knowledge scale (Jach et al., 2017) asks teens to rate the degree to which their parents see and understand their strengths. ‘Strengths’ here are defined broadly to include “personality, ability, talents and skills.” They are non-specific and subjective, that is, particular strengths listed in some strengths taxonomies (e.g., love of learning, kindness, empathy, perseverance) are not included in the scale. Participants responded to items (e.g., “My parents see the things I do best”) on a 7-point scale ranging from 1 (*strongly disagree*) to 7 (*strongly disagree*). Scores ranged from 7 to 49.

The SBP knowledge scale has demonstrated strong internal consistency across several studies, with a current omega reliability coefficient of ω = 0.95, 95% CI [0.94,0.96]. The scale has shown discriminant validity from autonomy-granting and responsiveness parental styles, and incremental validity in predicting life satisfaction in teens over and above authoritative parenting (Waters, 2015b). The scale also shows moderate convergence between teen ratings of their parents’ strength knowledge and parents’ self-reported strength knowledge (Waters, 2015a).

#### Subjective Wellbeing

Following Diener (1984) model of subjective wellbeing, we assessed the three elements of positive affect (PA), negative affect (NA), and life satisfaction (LS). Positive and negative affect were measured using the 10-item shortened Positive and Negative Affect Schedule for Children (Ebesutani et al., 2012). Students rated the extent to which they felt positive affect (e.g., joyful, happy) and negative affect (e.g., miserable, afraid) on a 5-point scale ranging from 1 (*very slightly or not at all*) to 5 (*extremely*). Scores ranged from 5 to 50.

Life satisfaction was measured using the 5-item Satisfaction with Life Scale for Children (Gadermann et al., 2010). Participants responded to items (e.g., “In most ways my life is close to the way I would want it to be”) on a 5-point scale ranging from 1 (*disagree a lot*) to 5 (*agree a lot*). Convergent and discriminant validity has been demonstrated for these three components of subjective wellbeing in several other studies (Gadermann et al., 2010; Ebesutani et al., 2012). Scores ranged from 5 to 25.

### Data Analysis

Two statistical approaches to model longitudinal data were applied. Both use a structural-equation modeling (SEM) approach with latent variables indicated by survey items, thereby accounting for measurement error in scales. Latent factors also model change that occurs over time by fitting a curve to each participant’s scores, in what are commonly termed latent growth curve models (LGM; Duncan and Duncan, 2009), in order to examine univariate change in all study variables (hypothesis One and Two); and relationships between rates of growth between variables over the study period (hypothesis three). A random-intercept cross-lagged panel model (RI-CLPM; Hamaker et al., 2015) was also applied to test for possible within-person causal effects of SBP on PA, NA and LS (hypothesis Four) while controlling for and assessing the degree of between-person stability in the variables over time. As scale ranges vary across SBP and the other measures, we rely on reporting standardized (SD units) effects where suitable.

#### Latent Growth Models

An LGM investigated intra-individual change over time by modeling a latent trajectory for each participant’s three repeated measures. Scores at each time point were used as indicators of the trajectory (Raudenbush, 2001; Muthén and Muthén, 2015). With three data points, an LGM is limited to linear trajectories, but this technique can still be a useful way to examine within-person change and the correlation among change across multiple variables. In the present case, we investigated correlated change in SBP and LS, NA and PA simultaneously. The linear time factor included an additional 50% for the second lag, as the time difference between T2-T3 was approximately 50% longer (9 months vs. 6 months).

### Random Intercept Cross-Lagged Panel Model

RI-CLPM was to analyze causality between SBP and SWB. According to Hamaker et al. (2015) these models are suitable to non-experimental settings with datasets comprising three repeated measures or more. Like traditional cross-lagged panel models, the RI-CLPM construe causality similar to Granger (1988). However, the approach also apportions variance to a latent factor capturing a time-invariant average around which people’s levels on a given measure tend to regress over time. This approach facilitates examination of causal change (i.e., cross-lagged effects) separately to change associated with mean reversion (i.e., autoregression).

Longitudinal studies of life events and SWB highlight the importance of capturing stable within-person predispositions, which form early and are resistant to change in all but drastic events (Diener et al., 2018). The ability of the RI-CLPM to achieve this decomposition has been described by some as arising from separating within- and between-person variability (for an example with adolescent parental-related data see Keijsers, 2016). However, LGMs also undertake a similar ‘multi-level’ treatment of change over time by first modeling trajectories for individuals, then considering population distribution characteristics in the sample of these individual trajectories (Raudenbush, 2001). The autoregressive and cross-lagged paths in the RI-CLPM are reflective solely of within-person change, without the influence of between-person variance.

Initially, maximum-likelihood (ML) was the chosen estimator, but due to problems with convergence under ML, a Bayesian estimator with uninformative or diffuse prior probabilities was adopted – these priors allow Bayesian and ML results to converge in the long run (Muthén and Asparouhov, 2012). Specifically, under ML the RI-CLPM failed to converge in some cases due to very high latent correlations among between-person factors, indicative of strong correlations among stable traits driving stability in the variables. Bayesian estimators are known to perform better than ML under conditions where values are near the edges of their parameter spaces, as is the case here with highly correlated latent factors. For similar issues, Bayesian estimators are also becoming more widely applied in longitudinal analyses (Asparouhov et al., 2018). ML estimation was utilized for nested model tests of measurement and invariance properties. To investigate the influence of sample size on the key parameters of interest, a *post hoc* power analysis was undertaken by imputing the Bayesian-derived estimates into Monte Carlo simulations of varied sample sizes.

## Results

### Data Cleaning and Preparation

Data cleaning and preparation was undertaken in SPSS and Excel, with MPlus used for all substantive analyses. Data were examined for out-of-range values, survey completion times, open-ended comments that suggest spurious responses, duplicate I.P. addresses and/or participants, and missingness. One participant was cut due to a completion time of less than 2 min and many items in neutral. Fifteen cases had almost no items completed and were excluded. Only four other cases demonstrated some missingness, with approximately half the survey items missing in each case. These cases were retained as full-information- ML was utilized. Items were reverse-scored where necessary to consistently measure their underlying factor, and simple scale means calculated.

### Outliers

Data were screened for outliers. As shown in Table 1, univariate (±2 SD of the mean) and multivariate (Mahalanobis Distance, with probability from the Chi-Squared distribution greater than *p* < 0.001 with *df* = 12) outliers were identified in distributions of observed scores of the final matched sample (Tabachnick et al., 2001). Relatively few cases were identified as outliers, and a theoretical rationale for excluding univariate and multivariate outliers was not apparent. As such, all were retained.

**TABLE 1:** Distribution characteristics for observed variables.

**Variable**	**Observed**						
	
	***M***	***SD***	**Median**	**Mode**	**Skew**	**Kurt**	***n* univariate outlier (± 2 *SD*)**
SBP T1	39.02	9.14	42	49	−0.83	0.06	9 (all −)
SBP T2	38.27	9.87	40	49	−1.05	0.75	8 (all −)
SBP T3	37.02	8.52	38	42	−0.58	−0.21	8 (all −)
LS T1	18.37	4.65	19	19^∗^	−0.49	−0.52	8 (all −)
LS T2	18.09	4.43	18	17	−0.47	−0.29	8 (all −)
LS T3	17.06	4.23	17	20	−0.55	0.10	7 (all −)
PA T1	19.22	4.00	20	22	−0.78	0.41	10 (all −)
PS T2	18.44	3.84	19	19	−0.58	0.47	6 (all −)
PS T3	17.61	4.07	18	19^∗^	−0.71	0.42	2 + 8 −
NA T1	9.78	3.81	9	8	1.06	1.17	9 (all +)
NA T2	10.04	4.08	9	5	0.68	−0.28	6 (all +)
NA T3	10.69	4.07	10	10	0.67	−0.06	6 (all +)
Multivariate outliers *n* = 3 (Mahalanobis’ Distance, χ^2^, *p* < 0.001, *df* = 12)							

*SBP, Strength-based parenting knowledge. LS, life satisfaction. PA, positive affect. NA, negative affect. Skew, skewness. Kurt, kurtosis. Standard error of skewness = 0.17, standard error of kurtosis = 0.34. ^∗^ = smallest mode is shown. T denotes the wave of data collection. Total range for SBP is 7–49; NA, PA, and LS from 5 to 25.*

### Descriptive Statistics

Distributional characteristics for all variables are reported in Table 1 and correlations are presented in Table 2. Observed score distributions indicate acceptable levels of skewness and kurtosis, with SBP knowledge, LS, and PA somewhat negatively skewed (mean falling on the higher end of the scale), and NA positively skewed (mean falling on the lower end of the scale). A slight decrease in SBP simple scale mean scores was evident as time progressed, probabilistically indistinguishable from zero in T1-T2 (*M* = −0.11, *SD* = 1.23, *t* = −1.24, *p* = 0.22); a slightly larger and significant decrease from T2-T3 (*M* = −0.18, *SD* = 1.20, *t* = −2.12, *p* = 0.03); and a significant decline over the full study period (*M* = −0.29, *SD* = 1.34, *t* = −3.02, *p* = 0.01). Significant correlations were evident between SBP and SWB at all three time points. SBP was positively correlated with LS and PA, and inversely correlated with NA.

**TABLE 2:** Zero-order correlation matrix of all variables (observed, simple scale means).

**Variable**		**1**	**2**	**3**	**4**	**5**	**6**	**7**	**8**	**9**	**10**	**11**	**12**
1	SBP T1												
2	SBP T2	0.59^∗∗^											
3	SBP T3	0.44^∗∗^	0.59^∗∗^										
4	LS T1	0.45^∗∗^	0.38^∗∗^	0.33^∗∗^									
5	LS T2	0.32^∗∗^	0.49^∗∗^	0.39^∗∗^	0.66^∗∗^								
6	LS T3	0.32^∗∗^	0.36^∗∗^	0.46^∗∗^	0.54^∗∗^	0.59^∗∗^							
7	PA T1	0.40^∗∗^	0.26^∗∗^	0.21^∗∗^	0.62^∗∗^	0.48^∗∗^	0.36^∗∗^						
8	PA T2	0.23^∗∗^	0.35^∗∗^	0.20^∗∗^	0.39^∗∗^	0.60^∗∗^	0.39^∗∗^	0.51^∗∗^					
9	PA T3	0.12	0.06	0.24^∗∗^	0.21^∗∗^	0.27^∗∗^	0.40^∗∗^	0.36^∗∗^	0.34^∗∗^				
10	NA T1	–0.28^∗∗^	–0.22^∗∗^	−0.17^∗^	–0.46^∗∗^	–0.38^∗∗^	–0.32^∗∗^	–0.39^∗∗^	–0.31^∗∗^	−0.16^∗^			
11	NA T2	–0.21^∗∗^	–0.21^∗∗^	–0.20^∗∗^	–0.49^∗∗^	–0.61^∗∗^	–0.40^∗∗^	–0.41^∗∗^	–0.42^∗∗^	–0.13	0.59^∗∗^		
12	NA T3	−0.15^∗^	–0.08	0.01	–0.28^∗∗^	–0.23^∗∗^	–0.22^∗∗^	–0.22^∗∗^	−0.14^∗^	0.19^∗∗^	0.36^∗∗^	0.50^∗∗^	

*SBP, Strength-based parenting knowledge. LS, life satisfaction. PA, positive affect. NA, negative affect. *N* = 202. T denotes the wave, with T1 being the first measurement interval/baseline. ^∗^*p* < 0.05, ^∗∗^*p* < 0.01, ^∗∗∗^*p* < 0.001.*

### Sample Bias Due to Attrition

Returners for all three waves (*N* = 202) were compared to non-returners of either time 2 or 3 (*N* = 380–504 depending on measure). As mental health is known to decline over the teen years, baseline year level was included as a control variable. To retain the benefit of accounting for measurement error, a structural equation model tested the effect of attrition status (a dichotomous predictor) on the continuous latent outcome variables. In this instance, the effect of attrition reflects a probit regression estimate using weighted least squares, while all other estimates reflect linear regression of continuous, latent variables, utilizing full information maximum likelihood to handle missing data.

Results of the attrition analysis are presented in Table 3. Students in later year levels had significantly lower cross-sectional SBP, LS, PA and higher NA at T1. In addition, higher baseline year level was associated with lower PA at T2. Only one of the outcomes variables was significantly predicted by attrition status, with students reporting higher life satisfaction at T2 more likely to complete all three waves. Given that this was the only significant difference, across 4 variables across 3 points in time, between responders and non-responders we are confident that the results of the final sample are reflective of the fuller sample at baseline.

**TABLE 3:** Tests of sample bias due to attrition.

**Variable**	***N* non returners**	**Baseline Year level**		**Returner status**	
			
		**β**	***SE***	**β**	***SE***
SBP T1	499	–0.171^∗∗∗^	0.049	0.154	0.132
SBP T2	382	–0.085	0.058	0.242	0.145
LS T1	504	–0.170^∗∗∗^	0.033	0.045	0.087
LS T2	381	–0.033	0.036	0.206^∗^	0.091
PA T1	504	–0.178^∗∗∗^	0.028	0.063	0.075
PA T2	381	–0.122^∗∗∗^	0.033	0.058	0.083
NA T1	504	0.137^∗∗∗^	0.029	–0.007	0.078
NA T2	380	0.087	0.036	–0.076	0.088

*SBP, Strength-based parenting knowledge. LS, life satisfaction. PA, positive affect. NA, negative affect. T denotes the time point. Returners to all three waves of data were dummy coded as ‘1’ and non-returners as ‘0.’ *N* = 202 participants returned for all three waves.*

### Measurement and Invariance Testing

Before proceeding to the RI-CLPM, invariance testing was undertaken to ensure measurement properties of the scale were comparable over time. Measurement invariance testing consisted of modeling a latent factor at each wave for each measure, freely covaried and using ML estimation, with measurement properties evaluated against Hu and Bentler’s (1999) simulation-based fit indices, with Cheung and Rensvold’s (2002) recommendation of a <0.01 change in CFI was used as the key basis for proceeding. Table 4 presents results of the measurement invariance testing, which was then applied in the RI-CLPM models.

**TABLE 4:** Measurement and invariance testing.

**Model**	**χ^2^**	***df***	**CFI**	**TLI**	**ΔCFI**	**M.I. EPC (stdyx)**	**λ (std) range**	**Decision**
**SBP**								
M	359.884	168	0.961	0.951			0.414–0.922	Accept
FL	382.988	180	0.959	0.952	−0.002			Accept
**FL&FI**	**406.569**	**194**	**0.957**	**0.953**	**−0.004**			**Final**
**LS**								
M	144.508	75	0.961	0.945			0.603–0.884	Accept
FL	153.290	83	0.960	0.950	−0.001			Accept
FL&FI	192.457	93	0.944	0.937	−0.017^+^	I: −0.110:*LS2t3*		Reject
FL&PI: *LS2t3*	181.504	92	0.950	0.942	−0.011^+^	I: −0.106:*LS3t3*		Reject
**FL&PI: *LS2t3, LS3t3***	**172.238**	**91**	**0.954**	**0.947**	**−0.007**			**Final**
**PA**								
M	156.909	75	0.958	0.941			0.578–0.964	Accept
FL	181.678	83	0.949	0.935	−0.009	L: −0.124:*PA3t1*		Accept
PL: *PA3t3*			0.951		−0.007	L: −0.133:*PA5t3*		Reject
PL: *PA3t3*, *PA5t3*	167.779	81	0.955		−0.003			Reject
PL&FI	214.041	91	0.936	0.927	−0.022^+^	I: −0.093:*PA4t3*		Reject
PL&PI: *PA4t3*	203.826	90	0.941	0.931	−0.017^+^	I: −0.090:*PA3t3*		Reject
PL&PI: *PA4t3*, *PA3t3*	195.667	89	0.945	0.935	−0.013^+^	I: −0.075:*PA1t3*		Reject
**PL: *PA3t1, PA5t3*&PI: *PA4t3, PA3t3, PA1t3***	**186.543**	**88**	**0.949**	**0.939**	**−0.009**			**Final**
**NA**								
M	391.952	75	0.782	0.694			0.583–0.875	Accept
FL	404.596	83	0.779	0.720	−0.003			Accept
FL&FI	473.101	93	0.738	0.705	−0.044	I: 0.241:*NA2t3*		Reject
FL&PI: *NA2t3*	440.005	92	0.760	0.727	−0.022	I: −0.178:*NA5t3*		Reject
**FL&PI: *NA2t3, NA5t3***	**418.063**	**91**	**0.775**	**0.740**	**−0.007**			**Final**

*SBP, strength-based parenting knowledge. LS, life satisfaction. PA, positive affect. NA, negative affect. *df*, degrees of freedom. CFI, comparative fit index. TLI, Tucker Lewis index. M.I. EPC (stdyx), minimum expected parameter change, standardized. Model conventions: M, measurement. FL, full loading. FI, full intercept. PL, partial loading: *freed loading survey item(s)*. PI, partial invariance: *freed intercept survey item(s)*. *t*1-3 denotes survey wave. ^+^ a change in CFI of 0.01 or greater from the measurement model was adopted as the guideline for invariance acceptability. ΔCFI, change in CFI from the measurement model to the present model. Maximum likelihood estimator was utilized for measurement and invariance testing. For PA, while partial loading invariance held, the decrement in fit was close to 0.01 (ΔCFI = −0.007), and as such loadings were freed before proceeding to tests of intercept invariance. Final model adopted and utilized in all LGM and RI-CLPM models for each factor is in bold.*

Strength-based parenting had good measurement qualities and retained good fit under scalar invariance. SWL had good model fit indices under metric invariance, but fit declined under a scalar invariance, and a partial intercept invariance model was adopted, with LS items 3 and 2 intercepts being freely estimated. Possibly consistent with the more temporally variable nature of emotion, which is itself an indicator of healthy emotional function (Koval et al., 2016), PA and NA both demonstrated poorer invariance properties; and NA also had poorer single-factor measurement properties. PA had acceptable fit indices but required one factor loading to be freely estimated to retain good fit, and all but one intercept to be freely estimated. NA had poor measurement qualities, but without any very low loading items, or a theoretical rationale for modeling anything but a one-factor solution to the scale, invariance testing was not undertaken. Results of the RI-CLPM including NA should therefore be interpreted with caution. The final measurement models identified in Table 4 were utilized in the subsequent latent growth and random intercept cross-lagged panel models.

### Hypotheses One and Two: Latent Growth Curve Model

The LGM provides a robust test of intra-individual change across the sample in SBP and SWB and forms the tests of hypotheses 1–3. As the LGM controls for measurement error by design, simple scale mean scores were used, and to account for the unequal data collection waves an additional 50% of time was included in the time factor in the second lag. A single LGM simultaneously estimated univariate and correlated change in SBP, LS, PA, and NA.

This LGM was initially modeled with ML estimation, which provided good fit (χ^2^(34) = 119.934, *p* < 0.001; RMSEA.112 [0.091.134]; CFI.922; TLI.849), suggesting a linear curve fit the sample well. However, a non-positive definite matrix made the solution unreliable. As such, a Bayesian estimator with 10000 iterations and diffuse/uninformative priors was adopted, which converged according to acceptable criteria (a posterior scale reduction or PSR < 1.05 for the latter half of iterations). Posterior SD is reported as Standard Error (SE) for Bayesian-derived estimates.

### Hypothesis One

Latent growth-curve models results demonstrate a significant univariate decline in within-person SBP across the time period (unstandardized mean slope factor μ_SBP–S_ = −0.12. [−0.19 −0.04], SE/PSD (posterior SD) = 0.04, *p* < 0.001), thus hypothesis one was supported. Participants starting values also influenced their change over time. Covariances between the intercept and slope factors in the LGM indicate participants who began with initially higher scores tended to decline at a greater rate in SBP (unstandardized ψ_SBP__–I SBP__–S_ = −0.26 [−0.43 −0.16], *SE* = 0.07, *p* < 0.001).

### Hypothesis Two

In relation to wellbeing, a significant within-person decline in all components of SWB over the time period was found, thus supporting hypothesis two. A negative and significant mean latent trajectory was evident for LS (unstandardized μ_LS__–S_ = −0.11 [−0.16 −0.06, *SE* = 0.03, *p* < 0.001), and PA (unstandardized μ_PA–S_ = −0.13 [−0.18 −0.08], *SE* = 0.03, *p* < 0.01), and a positive trend for NA (unstandardized μ_NA–S_ = 0.074 [0.03.22], *SE* = 0.03, *p* < 0.01). As with SBP, participants starting values influenced their change over time. Covariances between the intercept and slope factors in the LGM indicate participants who began with initially higher scores tended to decline at a greater rate in PA (unstandardized ψ_PA–I PA–S_ = −0.09 [−0.15 −0.04], *SE* = 0.03, *p* < 0.001) and increase more for NA (unstandardized ψ_NA–__I NA–__S_ = −0.05 [−0.11, −0.01], *SE* = 0.03, *p* < 0.01).

### Hypothesis Three

The LGM also tested whether rates of change in SBP and aspects of SWB were related. A significant slope correlation between SBP and wellbeing variables was evident. Increases in SBP during the study period was associated with growth in LS (unstandardized B_SBP–S LS–S_ = 0.05 [0.02,0.079], *SE* = 0.02, *p* < 0.001) and PA (unstandardized B_SBP–S PA–S_ = 0.06 [0.03,0.09], *SE* = 0.02, *p* < 0.001); but not NA (standardized B_SBP–S NA–S_ = 0.01 [−0.03,0.03], *SE* = 0.02, *p* < 0.430); partially confirming hypothesis three.

### Hypothesis Four

**FIGURE 1:**
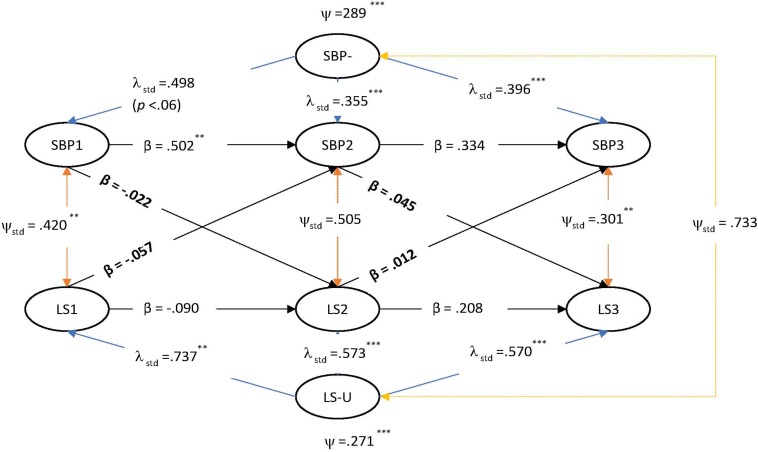
RI-CLPM of strength-based parenting and life satisfaction.

**FIGURE 2:**
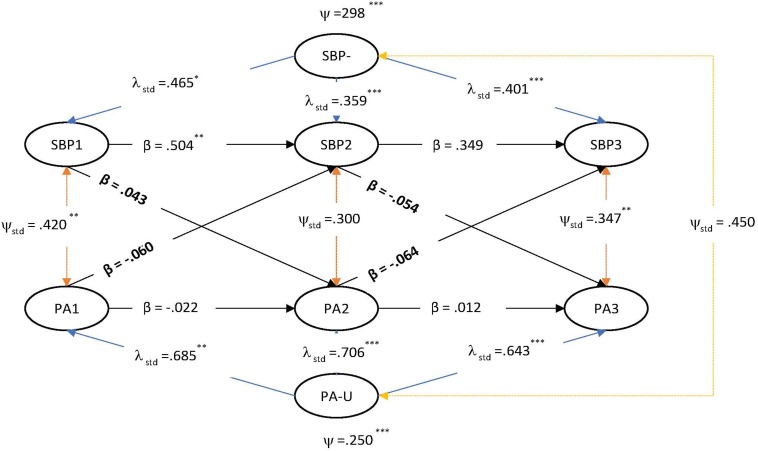
RI-CLPM of strength-based parenting and positive affect.

**FIGURE 3:**
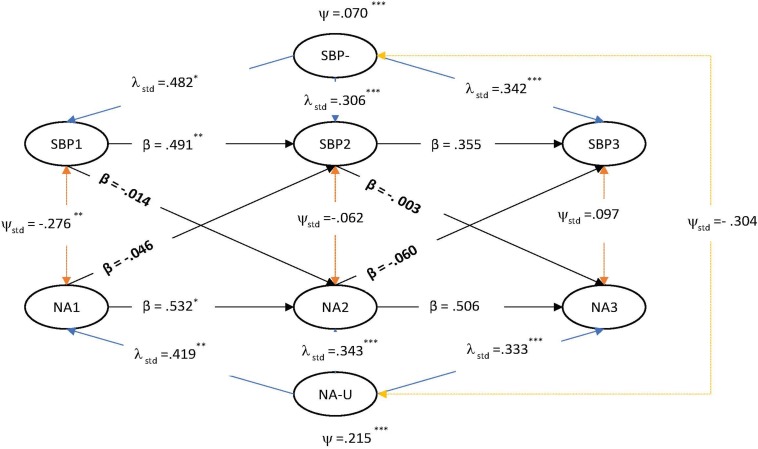
RI-CLPM of strength-based parenting and negative affect.

Three RI-CLPMs each tested the potential causal and reciprocal relationships between SBP and LS, PA and NA. Non-positive definite matrices were encountered using the ML estimator, and, as such, a Bayesian estimator with 10,000 iterations was utilized with default diffuse uninformative priors, as in the LGM. The final measurement models adopted were per invariance testing. Results of the RI-CLPMs are presented in Figures 1–3.

As with the LGM testing, the RI-CLPMs found significant positive contemporaneous effects between SBP with LS, PA and NA at time 1 and then again between SBP with LS and PA at time three. These results, described by some as “correlated change” (Keijsers, 2016), are in line with the LGM and also with past findings of the beneficial effects of SBP on teen wellbeing and suggest that SBP is important factor for teenager wellbeing in their immediate time frames (Loton and Waters, 2017).

In relation to the test of time-variant stability/instability, the random intercept was significant in each model for each variable, although smaller in the case of NA: SBP-U (in the LS model) = 0.29 [0.01 – 2.4], *SE* = 0.64, *p* < 0.001; LS-U = 0.26 [0.01 – 1.13], *SE* = 0.32, *p* < 0.001; PA-U = 0.24 [0.03 −0.69], *SE* = 0.54, *p* < 0.001; NA-U = 0.06 [0.01 −0.53], *SE* = 0.14, *p* < 0.001. The ICC for each between-variable (i.e., ‘random intercept’ latent trait variance/total average variance for each latent factor from times 1–3) indicate most variance was time-specific/within-person. The between-person proportions for SBP were 13.0%, LS = 24.7%, PA = 33.8%, and NA = 34.7%. In the present case, a participant’s level of SBP at time one did predict SBP at time two (∼6 months) but this effect was not retained over the second lag (∼9 months). These results are consistent with past research showing that parenting is characterized by stability *and* change (Holden and Miller, 1999).

The substantive parameters of interest in the RI-CLPM are the cross-lagged paths, which represent an indication of causal predominance, and are interpreted as ‘*predicting change’* at the within-person level (Hamaker et al., 2015, p. 104). In our models, all cross-lagged paths were not significant, pointing instead to possible ‘third factors’ that may explain the strong positive contemporaneous effects evident between SBP and SWB.

### Power Analysis

With a sample of *N* = 202 and 82 parameters freely estimated in the RI-CLPMs, the sample size to parameter ratio exceeds common guidelines (Bentler and Chou, 1987). However, the indicators to latent factor ratio is relatively high, which is sometimes thought to compensate for the detrimental effect of small samples on power in SEM (Marsh et al., 1998). As such, a *post hoc* power analysis was undertaken using Monte Carlo simulation (see Muthén and Muthén, 2002) of the Bayesian-derived estimates, to determine the increase in sample size required for CL effects to reach a significance level of *p* < 0.05. Results are presented in Table 5. Based on these simulation specifications, the CL path results held even in simulated samples up to *N* = 5002.

**TABLE 5:** *Post hoc* power analysis: Monte Carlo simulations of larger samples using Bayesian-derived estimates.

***N***	**Percentage of draws in which the cross-lagged path is significant at *p* < 0.05**			
	**SBP to LS lag 1**	**SBP to LS lag 2**	**LS to SBP lag 1**	**LS to SBP lag 2**
402	0.00%	0.01%	0.00%	0.00%
1002	0.00%	0.00%	0.00%	0.00%
5002	0.03%	0.00%	0.03%	0.01%
	**SBP to PA lag 1**	**SBP to PA lag 2**	**PA to SBP lag 1**	**PA to SBP lag 2**
402	0.00%	0.00%	0.00%	0.00%
1002	0.00%	0.04%	0.00%	0.00%
5002	0.03%	0.03%	0.03%	0.03%
	**SBP to NA lag 1**	**SBP to NA lag 2**	**NA to SBP lag 1**	**NA to SBP lag 2**
402	0.02%	0.00%	0.00%	0.00%
1002	0.00%	0.00%	0.02%	0.00%
5002	0.08%	0.03%	0.01%	0.03%

*SBP, strength-based parenting knowledge. LS, life satisfaction. PA, positive affect. NA, negative affect. Initial sample *N* = 202, repetitions = 500, seed = 45355, population and coverage both defined by the Bayesian RI-CLPM outputs.*

## Discussion

Early adolescence is a pivotal period of development and parents play a key role in the mental health of young people during this life stage (Bruyn et al., 2003; Galambos et al., 2003; Bøe et al., 2014; Steinberg, 2014). Understanding the role of positive parenting on teen SWB motivated the present study. With past evidence for the concurrent and two-wave repeated measures beneficial effects of SBP on teen wellbeing (Waters, 2015b; Loton and Waters, 2017; Waters et al., 2019), we extended the time frames to a three-wave, fourteen-month study. Based on findings in past studies in the field of parenting research, we hypothesized a decline in SWB (hypothesis one) and SBP (hypothesis two) together with a relationship and causal impact of SBP on SWB over time (hypotheses three and four respectively).

Three of the four hypotheses were supported. Firstly, as predicted, SWB diminished significantly over time, confirming hypothesis one. Specifically, LS and PA declined while NA increased over time. Secondly, within-person SBP significantly declined over the study period, supporting hypothesis two. Thirdly, teens who reported a decrease in SBP over the study period also tended to experience declines in LS and PA, but no change to the trajectory of NA, as revealed through the LGM analysis. The same beneficial contemporaneous effects of SBP on LS and PA, but not NA, were evident in correlated change at T1 and T3 through the RI-CLPM analyses, thus providing partial support hypothesis three. In regards to hypothesis four, we examined these dynamic relationships by applying a causal panel modeling framework that partitions variance into stable/time-invariant components and links within-person fluctuations across each wave for each variable (Hamaker et al., 2015). In these models, results were partially supportive of the SBP-SWB link but did not support direct causal effects. The cross-lagged paths indicated that within-person changes in SBP did not predict increases in SWB over time, or vice-versa, thus failing to support hypothesis four.

When the causal analysis framework was applied, the degree of stability across factors ranged from 13% to 34.7% of variance apportioned to between-person stability which highlights the importance of modeling change in a multi-level framework. In addition, rank-order stability in SBP held only in the first 6 but not over the ensuing 9 months, showing a pattern of variance-invariance in parenting, and supporting past research that positive parenting changes through the adolescent years.

The decline in wellbeing during early adolescence is highly consistent with past research (Ullman and Tatar, 2001; Suldo and Huebner, 2004; Park, 2005) and is likely to be a function of the significant life changes young people go through when stepping into the second decade of their lives. Developmental psychologists have long identified that the shift from childhood into adolescence marks a time of intense social, physical, identity and economic changes (Larson and Wilson, 2004; Sodha, 2009; Dorn and Biro, 2011). Together with increases in responsibilities and academic pressures, these changes put early adolescents at risk of mental ill-health (Frigerio et al., 2009; Anselmi et al., 2010).

Neuroscientists have also now added to our understanding of why mental health declines in this age bracket by showing that adolescence is a time where the brain is particularly vulnerable to stress and depression (Andersen and Teicher, 2008) because it is undergoing rapid change (Giedd, 2008), including the process of synaptic pruning of gray matter coupled with new production of white matter (Giedd, 2008). The ‘back to front’ development of brain structures in the teen years from the amygdala, through to the hippocampus and on to the prefrontal cortex lead to the situation where the emotional systems of the brain develop faster than the cognitive systems of the brain (Sowell et al., 2001) which has been posited as a reason for greater emotional sensitivity and vulnerability in the teen years. Ernst et al. (2006) hypothesized that adolescent depression may emerge because the limbic structures that drive negative emotions mature more quickly than the pre frontal cortext which assists teens to regulate their mood. Toward the end of adolescence and in early adulthood the brain becomes more integrated and connected (Paus et al., 2008), and the cognitive systems catch up with emotional systems which scientists suggest is a reason why the risk for psychopathology diminishes in early adulthood (Giedd, 2008).

Importantly, neuroscientists have also shown that the type of parenting that occurs in the life of a young person transitioning into early adolescence has a significant impact on mental health. For example, early teens whose parents had displayed contemptuous, angry, impatient, belligerent, disapproving, threatening, or argumentative behaviors during a lab experiment in their late childhood were more likely to experience the onset of depressive symptoms and major depression in adolescents (Yap et al., 2008; Schwartz et al., 2011). Conversely, early teens whose parents related to them in approving, validating, affectionate, humorous, happy, pleasant, and/or caring ways, showed beneficial brain growth in regions that supports a teenager’s capacity for social-emotional functioning as well as declines in areas that make an early adolescent vulnerable to mental illness^9^ (Whittle et al., 2014).

In line with the above research that highlights the importance of positive parenting for youth wellbeing, both the LGM and RI-CLPM analyses revealed a pattern of positive contemporaneous effects between SBP and SWB. When pre-teens and early teens are asked to reflect on the parenting they receive and, at the same time, report on their wellbeing, a significant relationship is present. These results are consistent with those found across multiple samples of teens where SBP has been related to a range of wellbeing indicators such as SWB (Jach et al., 2017), life satisfaction (Waters, 2015b), happiness and self-efficacy (Loton and Waters, 2017), subjective happiness (Sağkal and Özdemir, 2019), and family happiness (Waters, in press).

Waters has argued that having strength-based parents provides teens with an interpersonal context that supports the development and reinforcement of strengths (Waters, 2015b). In support of this, Jach et al. (2017) found that SBP increased teen’s use of their strengths. Other studies have shown that strengths-use and development is a significant factor in youth well-being (Proctor et al., 2011; Suldo et al., 2014). Adding to the interpersonal context triggered by SBP, Waters (2015a) proposed that SBP creates an intrapersonal trigger for positive self-identity because having knowledge of one’s strengths creates a lens through which teens engage with the world and, thus, a positive filter for teen identity. Related to this, past research has shown that SBP predicts positive aspects of teen identity such as self-efficacy (Loton and Waters, 2017) and mental toughness (Sağkal and Özdemir, 2019).

An important finding in the current study was the non-significant relationship between SBP and SWB over time. While the degree of SBP teens reported that they were receiving was linked to their levels of LS and PA in ‘real time’^10^ this real time relationship did not transfer to longer time frames. That is, SBP at time one was not predictive of SWB at time two or three (6 and 14 months later). Likewise, SBP at time two was not predictive of SWB at time three (9 months later). This finding differs to past studies that have found SBP significantly predicts life satisfaction 12 months later (Waters, 2015b) and academic performance 3 months later (Waters et al., 2019). However, these past studies did not employ the stringent statistical analysis of RI-CLPM.

One reasons why SBP may not predict future SWB for early adolescents could be the reduction in the parent–child closeness that occurs during this particular life stage. Certainly, it is well recognized that increased distance and separation between parent and child are characteristic of the age bracket targeted for the current study due to the psychological need for ‘individuation’ (Levy-Warren, 1999). Larson and Richards’ (1991) study of differences in time spent with parents between 5th and 9th graders found that early teens spent 40% less time with their parents than those in their late childhood. This life stage also marks a period of emotional distance and research shows that reductions in parent-child closeness together with increases in child secrecy are especially prominent in the early adolescent years (Larson and Richards’, 1991; Keijsers et al., 2010). Steinberg and Silverberg (1986) assert that young teens no longer allow their parents to know everything about their lives. The process of separation and distance that unfolds during early adolescence may explain why parenting approaches that are present in the beginning of this study (e.g., pre-teens) are not having an impact on the wellbeing of the young person 14 months later because the relationship is become more distant. Future researchers could include measures of teen-parents closeness and conflict to test for the potential mediating or moderating effects of these two variables on changes to teen ratings of SBP over time. It might also be fruitful for future researcher to collect data about SBP from teen-parent dyads or triads (see Waters, 2015b) to examine the way parent-teen perceptions of SBP interact and change over time. The degree to which parent-teen perceptions of SBP get more aligned or more discrepant over time might influences psychological outcomes such as subjective wellbeing.

Whatever the cause, the results of this study indicate that parents cannot assume that their prior, or current, levels of SBP are ‘banked’ by their children to support future wellbeing. Instead, SBP needs to be a frequent, ongoing approach. Indeed, the fact that SBP was related to LS and PA in real time in our sample suggests that parents can contribute to the wellbeing of their sons and daughters at each step along the way by committing to regularly helping their pre-teens and teens cultivate their strengths.

Despite the importance of SBP, it was not consistent over time in current study with the youth samples’ ratings of SBP declining. The current findings are similar to past research showing a decline in a range of positive aspects of parenting such as parental warmth, support, involvement, and regard (McGue et al., 2005; Hafen and Laursen, 2009). Therefore, the decline in SBP that we observed may be related to this particular life stage, and the changes in the parent–child relationship that occur during this time. Waters (2015b) argues that SBP provides interpersonal benefits but it may be that in the early adolescent phase, a phase marked by increased parent-teen conflict, being strength-based is more difficult to achieve. The negative emotions that arise through conflict may heighten certain cognitive biases, such as the negativity bias in either the parent or the teen (Baumeister et al., 2001; Robin and Foster, 2002), making them focus more on problems and challenges rather than strengths and opportunities.

The reduction in SBP may also be to do with intrapersonal changes that occur for teens during this stage, especially the identity changes and increases in self-doubt. Klimstra et al. (2010) found that identity uncertainty increased in early to-middle adolescence. Increased self-doubt in the teen may mean that, even if the parent remains constant in their strength-based approach, the teen is not able to consistently absorb and integrate the positive feedback, thus accounting for reports of declines in SBP.

### Study Limitations and Future Research

This study has a number of positive features. It provides an example of a thorough analysis of observational panel data examining the relationship between parenting and wellbeing in an important life phase - early adolescence. The rigorous statistical modeling using both LGM and RI-CLPM are a strength of this study and encourage future researchers to ensure that the nuanced changes in phenomena over time are adequately tested. The measures were psychometrically sound and had been used in prior research. The sample was drawn from a socio-economically typical school rather than a focus on at-risk students allowing us to examine an under-explored strata of families. The study took an asset-based approach and adds to the small, but growing, literature on positive parenting, thus responding to the criticism of the parenting literature being overly deficit-oriented.

Alongside the above assets of the paper, we must also recognize several weaknesses. Firstly, the study was only able to provide a view of how SBP and SWB changed over 14 months. Given that early adolescence stretches over 3 years the study may have found different results had we extended the time lines. Perhaps SBP may have been causally linked with SWB had we followed the sample for longer into the next stage of adolescence where parents and their teens become closer again.

Secondly, the time points of data collection within the 14-month timeframe may have been too long to meaningfully examine causality. Six months and nine months are a long time in the life of a teen whose minds track time differently to adults (Steinberg, 2008). The current study suggested that temporal dynamics between SBP and SWB could instead operate in short timeframes, and future research may choose to use methods that tap more into the ‘real time’ dynamics such as daily diary methods (Fisher and Gershuny, 2013) or experiential sampling methods (Hektner et al., 2006) in order to better explore the mechanisms that are underpinning the SBP-SWB link.

Thirdly, the small sample size relative to the large number of estimated factors is another limitation, although a Monte-Carlo analysis simulating larger samples sizes with identical parameter solutions supported no change on that basis to the results. The use of Bayesian rather than ML estimators in the present study, increasingly used in dynamic examinations (Asparouhov et al., 2018), provides an example for social scientists wanting to model complex factors in small samples over time. Future similarly designed studies with larger samples are required to confirm the present results.

## Conclusion

Seligman and Csikszentmihalyi (2000) assert that “promoting competence in children is more than fixing what is wrong with them. It is about identifying and nurturing their strongest qualities, what they own and are best at, and helping them find niches in which they can best live out these strengths” (p. 6). The current study is relevant for professionals working with parents and prompts them to encourage parents to maintain a strength-based approach over time, so as to avoid the more typical patterns of parent-child decline in early adolescence. The study offers several opportunities for future research on this topic and we hope to be part of an ever-growing movement toward the study and practice of positive parenting.

## Ethics Statement

This study was approved by The University of Melbourne Human Research Ethics Committee, application number 1748708.

## Author Contributions

LW drafted and substantially edited the literature and discussion. DL and MZ undertook the analysis and wrote the “Materials and Methods” and “Results” section. DL assisted in drafting the introduction and substantially drafted the first draft of the discussion. DG reviewed the literature and drafted most of the first draft of the introduction. RJ-H assisted with critical feedback and data preparation.

## Conflict of Interest

LW has published a book on strength-based parenting and has been a speaker, including as an invited keynote, both *pro bono* and remunerated, at a number of conferences speaking on the topic of strength-based parenting. LW is co-founder of The Strengths Exchange, a website offering free strength-based resources and tools to parents (http://www.the-strengths-exchange.com.au). The remaining authors declare that the research was conducted in the absence of any commercial or financial relationships that could be construed as a potential conflict of interest.

## Footnotes

1 It is important to note that Costello et al. (2011) found a decrease in separation anxiety disorder and attention-deficit hyperactivity disorder from childhood into the teen years.

2 www.viacharacter.org

3 www.gallupstrengthscenter.com

4 blog.cappeu.com/realise2/

5 Note: autonomy granting parenting has also been studied in at risk and troubled youth.

6 The current study focuses on variance-invariance over time but it is also important to note that the stability of parenting has also been examined across contexts, tasks and siblings (see Holden and Miller, 1999; Madigan et al., 2016).

7 Note: The large majority of the studies included in Holden and Miller’s meta-analysis were of parenting in the first year of a baby’s life (45%), followed by studies of parenting from ages 1–3 (32%). This meta-analysis included only four studies that contained teenage sample (7%).

8 Rodríguez et al. (2014) found gender differences in parenting. Modest declines in *paternal* warmth were evident from early to late adolescence, but *maternal* warmth was high and stable across this time period.

9 In this neuroscientific study, positive maternal behavior was linked to accelerated cortical thinning in left and right orbitofrontal cortices, between baseline and follow up. Thinning of the orbitofrontal cortices is associated with superior cognitive functioning. For boys there was also a thinning in the right anterior cingulate. Thinning of the orbitofrontal cortices is associated with superior cognitive functioning. Thinning of the orbitofrontal cortices and anterior cingulate cortices is associated with higher temperamental effortful control (i.e., self-regulation and impulse control) and lower levels of internalizing behaviors (e.g., depression and anxiety). This research also found reduced growth of the right amygdala. Reduced growth in the amygdala is associated with a lower fear response, lower emotional reactivity and higher emotional regulation abilities.

10 The significant relationship was found at the baseline and 14 month re-tests.

## References

[B1] Andersen S. L. Teicher M. H. (2008). Stress, sensitive periods and maturational events in adolescent depression. *Trends Neurosci.* 31 183–191. 10.1016/j.tins.2008.01.004 18329735

[B2] Anselmi L. Fleitlich-Bilyk B. Menezes A. M. Araujo C. L. Rohde L. A. (2010). Prevalence of psychiatric disorders in a Brazilian birth cohort of 11-year-olds. *Soc. Psychiatr. Psychiatr. Epidemiol.* 45 135–142. 10.1007/s00127-009-0052-2 19381426

[B3] Asparouhov T. Hamaker E. L. Muthén B. (2018). Dynamic structural equation models. *Struct. Equ Modeling* 25 359–388. 10.1080/10705511.2017.1406803

[B4] Barnard M. McKeganey N. (2004). The impact of parental problem drug use on children: what is the problem and what can be done to help? *Addiction* 99 552–559. 10.1111/j.1360-0443.2003.00664.x 15078229

[B5] Baumeister R. Bratslavsky E. Finkenauer C. Vohs K. D. (2001). Bad is stronger than good. *Rev. Gen. Psychol.* 5 323–370. 10.1037/1089-2680.5.4.323

[B6] Baumrind D. (1991a). The influence of parenting style on adolescent competence and substance use. *J. Early Adolesc.* 11 56–95. 10.1177/0272431691111004

[B7] Baumrind D. (1991b). “Parenting styles and adolescent development,” in *The Encyclopedia on Adolescence*, eds Brooks-Gunn J. Lerner R. Petersen A. C. (New York, NY: Garland.), 746–758.

[B8] Bentler P. M. Chou C. H. (1987). Practical issues in structural modeling. *Soc. Methods Res.* 16 78–117. 10.1177/0049124187016001004

[B9] Bentley T. Widom C. S. (2012). A 30-year follow-up of the effects of child abuse and neglect on obesity in adulthood. *Obesit* 17 1900–1905. 10.1038/oby.2009.160 19478789

[B10] Biswas-Diener R. Kashdan T. B. Lyubchik N. (2017). “Psychological Strengths at work,” in *The Wiley Blackwell Handbook of the Psychology of Positivity and Strengths-Based Approaches at Work*, eds Oades L. G. Steger M. Delle Fave A. Passmore J. (West Sussex: Wiley Blackwell.), 34–47. 10.1002/9781118977620.ch3

[B11] Bøe T. Øverland S. Lundervold A. J. Hysing M. (2012). Socioeconomic status and children’s mental health: results from the bergen child Study. *Soc. Psychiatry Psychiatr. Epidemiol.* 47 1557–1566. 10.1007/s00127-011-0462-9 22183690

[B12] Bøe T. Serlachius A. S. Sivertsen B. Petrie K. J. Hysing M. (2018). Cumulative effects of negative life events and family stress on children’s mental health: the bergen child study. *Soc. Psychiatry Psychiatr. Epidemiol.* 53 1–9. 10.1007/s00127-017-1451-4 29090324

[B13] Bøe T. Sivertsen B. Heiervang E. Goodman R. Lundervold A. J. Hysing M. (2014). Socioeconomic status and child mental health: the role of parental emotional well-being and parenting practices. *J. Abnorm. Child Psychol.* 42 705–715. 10.1007/s10802-013-9818-9 24150864

[B14] Bor W. Dean A. J. Najman J. Hayatbakhsh R. (2014). Are child and adolescent mental health problems increasing in the 21st century? A systematic review. *Aust. N. Z. J. Psychiatry* 48 606–616. 10.1177/0004867414533834 24829198

[B15] Bornstein M. H. Tamis-LeMonda C. S. Hahn C.-S. Haynes O. M. (2008). Maternal responsiveness to young children at three ages: longitudinal analysis of a multidimensional, modular, and specific parenting construct. *Dev. Psychol.* 44 867–874. 10.1037/0012-1649.44.3.867 18473650

[B16] Brown B. (2004). “Adolescents’ relationships with peers,” in *Handbook of adolescent psychology*, eds Lerner R. M. Steinberg L. (Hoboken, NJ: Wiley), 331–361.

[B17] Brown J. Cohen P. Johnson J. G. Smailes E. M. (1999). Childhood abuse and neglect: specificity of effects on adolescent and young adult depression and suicidality. *Child Adolesc. Psychiatry* 38 1490–1496. 10.1097/00004583-199912000-00009 10596248

[B18] Bruyn M. D. Dekovic M. Meijnen G. W. (2003). Parenting, goal orientations, classroom behaviour and school success in early adolescence. *J. Appl. Dev. Psychol.* 24 393–412. 10.1016/s0193-3973(03)00074-1

[B19] Caprara G. V. Regallia C. Bandura A. (2002). Longitudinal impact of perceived self-regulatory efficacy on violent conduct. *Eur. Psychol.* 7 63–69. 10.1027/1016-9040.7.1.63

[B20] Cartwright-Hatton S. McNally D. Field A. P. Rust S. Laskey B. Dixon C. (2011). A new parenting-based group intervention for young anxious children: results of a randomized controlled trial. *J. Am. Acad. Child Adolesc. Psychiatry* 50 242–251. 10.1016/j.jaac.2010.12.015 21334564

[B21] Cheung G. W. Rensvold R. B. (2002). Evaluating goodness-of-fit indexes for testing measurement invariance. *Struct. Equ. Modeling* 9 233–255. 10.1207/S15328007SEM0902_5 22551991

[B22] Collins W. A. (1990). “Parent-child relationships in the transition to adolescence: COntinuity and change in interaction, affect, and cognition,” in *Advances in adolescent development: An annual book series, Vol. 2. From childhood to adolescence: A transitional period?*, eds Montemayor R. Adams G. R. Gullotta T. P. (Thousand Oaks, CA: Sage), 85–106.

[B23] Collishaw S. Maughan B. Goodman R. Pickles A. (2004). Time trends in adolescent mental health. *J. Child Psychol. Psychiatry* 45 1350–1362. 10.1111/j.1469-7610.2004.00335.x 15482496

[B24] Collishaw S. Maughan B. Natarajan L. Pickles A. (2010). Trends in adolescent emotional problems in England: a comparison of two national cohorts twenty years apart. *J. Child Psychol. Psychiatry* 51 885–894. 10.1111/j.1469-7610.2010.02252.x 20497281

[B25] Conger R. D. Conger K. J. Elder G. H. Jr. Lorenz F. O. Simons R. L. Whitbeck L. B. (1992). A family process model of economic hardship and adjustment of early adolescent boys. *Child Dev.* 63 526–541. 10.1111/j.1467-8624.1992.tb01644.x 1600820

[B26] Conoley C. W. Plumb E. W. Hawley K. J. Spaventavancil K. Z. (2015). Integrating positive psychology into family therapy: positive family therapy. *Couns. Psychol.* 43 703–733.

[B27] Costello E. J. Copeland W. Angold A. (2011). Trends in psychopathology across the adolescent years: what changes when children become adolescents, and when adolescents become adults? *J. Child Psychol. Psychiatry* 52 1015–1025. 10.1111/j.1469-7610.2011.02446.x 21815892 PMC3204367

[B28] Costello E. J. Mustillo S. Erkanli A. Keeler G. Angold A. (2003). Prevalence and development of psychiatric disorder in childhood and adolescence. *Arch. Gen. Psychiatry* 60 837–844. 12912767 10.1001/archpsyc.60.8.837

[B29] Costello J. E. Erkanli A. Angold A. (2006). Is there an epidemic of child or adolescent depression? *J. Child Psychol. Psychiatry* 47 1263–1271. 10.1111/j.1469-7610.2006.01682.x 17176381

[B30] Crone E. A. Dahl R. E. (2012). Understanding adolescence as a period of social-affective engagement and goal flexibility. *Nat. Rev. Neurosci.* 13 636–650. 10.1038/nrn3313 22903221

[B31] De Girolamo G. Dagani J. Purcell R. Cocchi A. McGorry P. D. (2012). Age of onset of mental disorders and use of mental health services: needs, opportunities and obstacles. *Epidemiol. Psychiatr. Sci.* 21 47–57. 10.1017/s2045796011000746 22670412

[B32] De Goede I. Barnie S. Meeus W. (2009). Developmental changes in adolescents’ perceptions of relationships with their parents. *J. Youth Adolesc.* 38 75–88. 10.1007/s10964-008-9286-7 19636793

[B33] de Graaf I. Speetjens P. Smit F. de Wolff M. Tavecchi L. (2008). Effectiveness of the Triple P positive parenting program on behavioral problems in children: a meta-analysis. *Behav. Modif.* 32 714–735. 10.1177/0145445508317134 18475003

[B34] Diener E. (1984). Subjective well-being. *Psychol. Bull.* 95 542–575. 6399758

[B35] Diener E. Oishi S. Tay L. (2018). Advances in subjective well-being research. *Nat. Hum. Behav.* 2 235–260. 10.1038/s41562-018-0307-6 30936533

[B36] Donaldson S. I. Dollwet M. Rao M. A. (2015). Happiness, excellence, and optimal human functioning revisited: examining the peer-reviewed literature linked to positive psychology. *J. Posit. Psychol.* 10 185–195. 10.1080/17439760.2014.943801

[B37] Dorn L. D. Biro F. M. (2011). Puberty and its measurement: a decade in review. *J. Res. Adolesc.* 21 180–195. 10.1111/j.1532-7795.2010.00722.x

[B38] Duan W. Ho S. Tang X. Li T. Zhang Y. (2014). Character strength-based intervention to promote satisfaction with life in the Chinese university context. *J. Happiness Stud.* 6 1347–1361. 10.1007/s10902-013-9479-y

[B39] Dubowitz H. Bennet S. (2007). Physical abuse and neglect of children. *Lancet* 369 1891–1899. 17544770 10.1016/S0140-6736(07)60856-3

[B40] Dumas J. E. (2005). Mindfulness-based parent training: strategies to lessen the grip of automaticity in families with disruptive children. *J. Clin. Child Adolesc. Psychol.* 34 779–791. 10.1207/s15374424jccp3404_20 16232075

[B41] Duncan T. E. Duncan S. C. (2009). The ABC’s of LGM: an introductory guide to latent variable growth curve modelling. *Soc. Pers. Psychol. Compass* 3 979–991. 10.1111/j.1751-9004.2009.00224.x PMC2888524 20577582

[B42] Dunst C. J. Deal A. G. (1994). “A family-centered approach to developing individualized family support plans,” in *Supporting and strengthening families: Methods, strategies, and practices*, eds Dunst C. J. Trivette C. M. Deal A. G. (Cambridge, MA: Brookline Books), 73–88.

[B43] Ebesutani C. Regan J. Smith A. Reise S. Higa-McMillan C. Chorpita B. F. (2012). The 10-Item positive and negative affect schedule for children, child and parent shortened versions: application of item response theory for more efficient assessment. *J. Psychopathol. Behav. Assess.* 34 191–203. 10.1007/s10862-011-9273-2

[B44] Erel O. Burman B. (1995). Interrelatedness of marital relations and parent-child relations: a meta-analytic review. *Psychol. Bull.* 118 108–132. 10.1037/0033-2909.118.1.108 7644602

[B45] Ernst M. Pine D. S. Hardin M. (2006). Triadic model of the neurobiology of motivated behavior in adolescence. *Psychol. Med.* 36 299–312. 10.1017/s0033291705005891 16472412 PMC2733162

[B46] Eyre O. Thapar A. (2014). Common adolescent mental disorders: transition to adulthood. *Lancet* 383 1366–1368. 10.1016/S0140-6736(13)62633-1 24439296

[B47] Farrant B. M. Devine T. A. J. Maybery M. T. Fletcher J. (2011). Empathy, perspective taking and prosocial behaviour: the importance of parenting practices. *Infant Child Dev.* 21 175–188. 10.1002/icd.740

[B48] Feldman M. (1994). Parenting education for parents with intellectual disabilities: a review of outcome studies. *Res. Dev. Disabi.* 15 299–332. 10.1016/0891-4222(94)90009-4 7972968

[B49] Feldman S. Elliott G. I. R. I. (eds) (1990). *At the Threshold: The Developing Adolescent.* Cambridge, MA: Harvard University Press.

[B50] Fisher K. Gershuny J. (2013). *Time Use and Time Diary Research.* Oxford: Oxford University Press.

[B51] Frigerio A. Rucci P. Goodman R. Ammaniti M. Carlet O. Cavolina P. (2009). Prevalence and correlates of mental disorders among adolescents in Italy: the PrISMA study. *Eur. Child Adolesc. Psychiatry* 18 217–226. 10.1007/s00787-008-0720-x 19165539

[B52] Gadermann A. M. Schonert-Reichl K. A. Zumbo B. D. (2010). Investigating validity evidence of the satisfaction with life scale adapted for children. *Soc. Indic. Res.* 96 229–247. 10.1007/s11205-009-9474-1

[B53] Galambos N. L. Barker E. T. Almeida D. M. (2003). Parents do matter: trajectories of change in externalizing and internalizing problems in early adolescence. *Child Dev.* 74 578–594. 10.1111/1467-8624.7402017 12705574

[B54] Geurtzen N. Scholte R. H. J. Engels R. C. M. E. Tak Y. R. van Zundert R. M. P. (2015). Association between mindful parenting and adolescents’ internalising problems: non-judgmental acceptance of parenting as core element. *J. Child Family Stud.* 24 1117–1128. 10.1007/s10826-014-9920-9

[B55] Govindji R. Linley P. A. (2007). Strengths use, self-concordance and well-being: implications for strengths coaching and coaching psychologists. *Int. Coach. Psychol. Rev.* 2 143–153.

[B56] Granger C. W. (1988). Some recent development in a concept of causality. *J. Econ.* 39 199–211. 10.1016/0304-4076(88)90045-0

[B57] Gray M. R. Steinberg L. (1999). Unpacking authoritative parenting: reassessing a multidimensional construct. *J. Marriage Fam.* 61 574–587.

[B58] Hafen C. A. Laursen B. (2009). More problems and less support: early adolescent adjustment forecasts changes in perceived support from parents. *J. Fam. Psychol.* 23 193–202. 10.1037/a0015077 19364213 PMC2754764

[B59] Hamaker E. L. Kuiper R. M. Grasman R. P. (2015). A critique of the cross-lagged panel model. *Psychol. Methods* 20 102–106. 10.1037/a0038889 25822208

[B60] Havighurst S. S. Wilson K. R. Harley A. E. Prior M. R. Kehoe C. (2010). Tuning in to kids: improving emotion socialization practices in parents of preschool children—findings from a community trial. *J. Child Psychol. Psychiatry* 51 1342–1350. 10.1111/j.1469-7610.2010.02303.x 20735794

[B61] Hawton K. Saunders K. E. O’Connor R. C. (2012). Self-harm and suicide in adolescents. *Lancet* 379 2373–2382. 10.1016/S0140-6736(12)60322-5 22726518

[B62] Hektner J. M. Schmidt J. A. Csikszentmihalyi M. (eds) (2006). *Experience Sampling Method: Measuring the Quality of Everyday Life.* Thousand Oaks, CA: Sage Publications, Inc.

[B63] Holden G. W. (1997). *Developmental Psychology Series. Parents and the Dynamics of Child Rearing.* Boulder, CO: Westview Press.

[B64] Holden G. W. Miller P. C. (1999). Enduring and different: a meta-analysis of the similarity in parents’ child rearing. *Psychol. Bull.* 125 223–254. 10.1037//0033-2909.125.2.223 10087937

[B65] Holmes S. J. Robins L. N. (1988). The role of parental disciplinary practices in the development of depression and alcoholism. *Psychiatry* 51 24–36. 10.1080/00332747.1988.11024377 3368544

[B66] Hu L. Bentler P. M. (1999). Cutoff criteria for fit indexes in covariance structure analysis: conventional criteria versus new alternatives. *Struct. Equ. Modeling* 6 1–55. 10.1080/10705519909540118

[B67] Huppert F. A. Abbott R. A. Ploubidis G. B. Richards M. Kuh D. (2010). Parental practices predict psychological well-being in midlife: life-course associations among women in the 1946 British birth cohort. *Psychol. Med.* 40 1507–1518. 10.1017/S0033291709991978 19995477 PMC3204412

[B68] Hussain A. Kumar A. Husain A. (2008). Academic stress and adjustment among high school students. *J. Indian Acad. Appl. Psychol.* 34 70–73.

[B69] Jach H. K. Sun J. Loton D. Chin T.-C. Waters L. (2017). Strengths and subjective wellbeing in adolescence: strength-based parenting and the moderating effect of mindset. *J. Happiness Stud.* 19 1–20. 10.1007/s10902-016-9841-y

[B70] Kaplan S. J. Pelcovitz D. Labruna V. (1999). Child and adolescent abuse and neglect research: a review of the Past 10 Years. Part I: physical and emotional abuse and neglect. *J. Am. Acad Child Adolesc. Psychiatry* 38 1214–1222. 10.1097/00004583-199910000-00009 10517053

[B71] Katsikitis M. Bignell K. Rooskov N. Elms L. Davidson G. (2013). The family strengthening programme: influences on parental mood, parental sense of competence and family functioning. *Adv. Men. Health* 11 143–151. 10.5172/jamh.2013.11.2.143

[B72] Katsikitis M. Jones C. Muscat M. Crawford K. (2014). Knowing You, Knowing Me (KYKM): an interactive game to address positive mother-daughter communication and relationships. *Front. Psychol.* 5:721. 10.3389/fpsyg.2014.0072 25071682 PMC4093658

[B73] Kehoe C. E. Havighurst S. S. Harley A. E. (2013). Tuning in to teens: improving parent emotion socialization to reduce youth internalizing difficulties. *Soc. Dev.* 23 413–431. 10.1111/sode.12060

[B74] Keijsers L. (2016). Parental monitoring and adolescent problem behaviors: how much do we really know? *Int. J. Behav. Dev.* 40 271–281. 10.1177/0165025415592515

[B75] Keijsers L. Branje S. J. T. Frijns T. Finkenauer C. Meeus W. (2010). Gender differences in keeping secrets from parents in adolescence. *Dev. Psychol.* 46 293–298. 10.1037/a0018115 20053026

[B76] Keijsers L. Poulin F. (2013). Developmental changes in parent–child communication throughout adolescence. *Devel. Psychol.* 49 2301–2308. 10.1037/a0032217 23477535

[B77] Kessler R. C. Avenevoli S. Merikangas K. R. (2001). Mood disorders in children and adolescents: an epidemiologic perspective. *Biol. Psychiatry* 49 1002–1014. 10.1016/S0006-3223(01)01129-5 11430842

[B78] Kieling C. Baker-Henningham H. Belfer M. Conti G. Ertem I. Omigbodun O. (2011). Child and adolescent mental health worldwide: evidence for action. *Lancet* 378 1515–1525. 10.1016/S0140-6736(11)60827-1 22008427

[B79] Kinard E. M. Klerman L. V. (1980). Teenage parenting and child abuse: are they related? *Am. J. Orthopsychiatry* 50 481–488. 10.1111/j.1939-0025.1980.tb03307.x 7406032

[B80] Kirby J. N. (2017). “Compassion-Focused Parenting,” in *The Oxford Handbook of Compassion Science*, eds Seppla E. M. Simon-Thomas E. Brown S. L. Worline M. C. Cameron C. D. Doty J. R. (Oxford: Oxford University Press), 91–105.

[B81] Kirby J. N. Baldwin S. (2018). A randomized micro-trial of a loving kindness meditation to help parents respond to difficult child behaviour vignettes. *J. Child Fam. Stud.* 27 1614–1628. 10.1007/s10826-017-0989-9

[B82] Klimstra T. A. Hales W. W. Raaijmakers Q. A. W. Branhe S. J. T. Meeus W. H. J. (2010). Identity formation in adolescence: change or stability? *J. Youth Adolesc.* 39 150–162. 10.1007/s10964-009-9401-4 20084561 PMC2807933

[B83] Koval P. Sütterlin S. Kuppens P. (2016). Emotional inertia is associated with lower well-being when controlling for differences in emotional context. *Front. Psychol.* 6:1997. 10.3389/fpsyg.2015.01997 26779099 PMC4705270

[B84] Kristjánsson K. (2012). Positive psychology and positive education: Old wine in new bottles? *Educ. Psychol.* 47 86–105. 10.1080/00461520.2011.610678

[B85] Larson R. Richards’ M. H. (1991). Daily companionship in late childhood and early adolescence: changing developmental Contexts. *Child Dev.* 62 284–300. 10.1111/j.1467-8624.1991.tb01531.x 2055123

[B86] Larson R. Wilson S. (2004). “Adolescence across place and time: Globalization and the changing pathways to adulthood,” in *Handbook of adolescent Psychology*, eds Lerner R. Steinberg L. (New York, NY: Wiley).

[B87] Larson R. W. Richards M. H. Moneta G. Holmbeck G. Duckett E. (1996). Changes in adolescents’ daily interactions with their families from ages 10 to 18: disengagement and transformation. *Dev. Psychol.* 32 744–754. 10.1037//0012-1649.32.4.744

[B88] Last A. Brownhill L. Ford T. Miles R. Wills L. (2012). Reliability and sensitivity to change of the family life questionnaire in a clinical population. *Child Adolesc. Men. Health* 17 121–125. 10.1111/j.1475-3588.2011.00621.x 32847297

[B89] Lerner R. M. Steinberg L. (eds) (2009). *Handbook(of)Adolescent Psychology, Volume 1: Individual Bases of Adolescent Development*, Vol. 1 New York, NY: John Wiley & Sons.

[B90] Levy-Warren M. H. (1999). “I am, you are, and so are we: a current perspective on adolescent separation–individuation theory,” in *Adolescent Psychiatry*, Vol. 24 ed. Esman A. H. (Hillsdale, NJ: The Analytic Press), 3–24.

[B91] Linley A. Willars J. Biswas-Diener R. (2010). *The Strengths Book: Be Confident, Be Successful, and Enjoy Better Relationships by Realizing the Best of You.* Coventry, CV: CAPP.

[B92] Loton D. Waters L. (2017). The mediating effect of self-efficacy in the connections between strength-based parenting, happiness and psychological distress in teens. *Front. Psychol.* 8:1707. 10.3389/fpsyg.2017.01707 29066986 PMC5641398

[B93] Lovejoy C. Graczyk P. O’Hare E. Neuman G. (2000). Maternal depression and parenting behavior: a meta-analytic review. *Clin. Psychol. Rev.* 20 561–592. 10.1016/s0272-7358(98)00100-7 10860167

[B94] Ma J. Han Y. Grogan-Kaylor A. Delva J. Castillo M. (2012). Corporal punishment and youth externalizing behaviour in Santiago, Chile. *Child Abuse Neglect* 36 481–490. 10.1016/j.chiabu.2012.03.006 22766372 PMC3493175

[B95] Maccoby E. (1984). Socialization and developmental change. *Child Dev.* 55 317–328. 10.1111/j.1467-8624.1984.tb00294.x

[B96] Maccoby E. E. (1992). Family structure and children’s adjustment: is quality of parenting the major mediator? *Monogr. Soc. Res. Child Dev.* 57 230–238. 10.1111/j.1540-5834.1992.tb00311.x

[B97] Madigan S. Plamondon A. Browne D. Jenkins J. (2016). Stability of observed maternal behaviour across tasks, time and siblings. *Parenting.* 16 108–124. 10.1080/15295192.2016.1134990

[B98] Marsh H. W. Hau K. T. Balla J. R. Grayson D. (1998). Is more ever too much? The number of indicators per factor in confirmatory factor analysis. *Multivariate Behav. Res.* 33 181–220. 10.1207/s15327906mbr3302_1 26771883

[B99] McArdle J. J. (2009). Latent variable modeling of differences and changes with longitudinal data. *Annu. Rev. Psychol.* 60 577–605. 10.1146/annurev.psych.60.110707.163612 18817479

[B100] McCloskey L. A. Figueredo A. J. Koss M. P. (1995). The effects of systemic family violence on children’s mental health. *Child Dev.* 66 1239–1261. 10.1111/j.1467-8624.1995.tb00933.x 7555214

[B101] McConachie H. Diggle T. (2007). Parent implemented early intervention for young children with autism spectrum disorder: a systematic review. *J. Eval. Clin. Pract.* 13 120–129. 10.1111/j.1365-2753.2006.00674.x 17286734

[B102] McCubbin H. I. Thompson E. A. Thompson A. I. Futrell J. A. (eds) (1999). *Resiliency in families, Vol. 4. The dynamics of resilient families.* Thousand Oaks, CA: Sage Publications, Inc.

[B103] McGue M. Elkins I. Walden B. Iacono W. G. (2005). Perceptions of the parent–adolescent relationship: a longitudinal investigation. *Dev. Psychol.* 41 971–984. 10.1037/0012-1649.41.6.971 16351340

[B104] Mejia A. Calam R. Sanders M. R. (2012). A review of parenting programs in developing countries: opportunities and challenges for preventing emotional and behavioral difficulties in children. *Clin. Child Fam. Psychol. Rev.* 15 163–175. 10.1007/s10567-012-0116-9 22427004

[B105] Mullen P. E. Martin J. L. Anderson J. C. Romans S. E. Herbison G. P. (1999). The long-term impact of the physical, emotional, and sexual abuse of children: a community study. *Child Abuse Neglect.* 20 7–21. 10.1016/0145-2134(95)00112-3 8640429

[B106] Muthén B. Asparouhov T. (2012). Bayesian structural equation modeling: a more flexible representation of substantive theory. *Psychol. Methods.* 17 313–335. 10.1037/a0026802 22962886

[B107] Muthén L. K. Muthén B. (2002). How to use a monte carlo study to decide on sample size and determine power. *Struct. Equ. Modeling* 9 599–620. 10.1207/s15328007sem0904_8

[B108] Muthén L. K. Muthén B. (2015). *Mplus: The Comprehensive Modelling Program for Applied Researchers: User’s Guide*, 5th Edn Los Angeles: Muthén & Muthén.

[B109] National Research Council (2011). “Toward an Integrated Science of Research on Families,” in *Workshop Report*, (Washington, D.C: National Academies Press). 21796829

[B110] Neff K. D. McGehee P. (2010). Self-compassion and psychological resilience among adolescents and young adults. *Self Identity* 2 225–240. 10.1080/15298860902979307

[B111] Park N. (2004). “Assessment and application,” in *Character Strengths and Virtues: A Handbook and Classification*, eds Peterson C. Seligman M. E. P. (Washington, DC: American Psychological Association), 6250644.

[B112] Park N. (2005). Life satisfaction among korean children and youth: a developmental perspective. *Sch. Psychol. Int. J.* 26 209–223. 10.1177/0143034305052914

[B113] Park N. Peterson C. (2006a). Character strengths and happiness among young children: content analysis of parental descriptions. *J. f Happiness Stud.* 7 323–341. 10.1007/s10902-005-3648-6

[B114] Park N. Peterson C. (2006b). Strengths of character and the family. *Fam. Ther. Mag.* 28–33.

[B115] Patton G. C. Coffey C. Romaniuk H. Mackinnon A. Carlin J. B. Degenhardt L. (2014). The prognosis of common mental disorders in adolescents: a 14-year prospective cohort study. *Lancet* 383 1404–1411. 10.1016/S0140-6736(13)62116-9 24439298

[B116] Patton G. C. Sawyer S. M. Santelli J. S. Ross D. A. Afifi R. Allen N. B. (2016). Our future: a lancet commission on adolescent health and wellbeing. *Lancet* 387 2423–2478. 27174304 10.1016/S0140-6736(16)00579-1 PMC5832967

[B117] Paus T. Keshavan M. Giedd J. N. (2008). Why do many psychiatric disorders emerge during adolescence? *Nat. Rev. Neurosci.* 9 947–957. 10.1038/nrn2513 19002191 PMC2762785

[B118] Peterson C. Seligman M. E. P. (2004). *Character Strengths and Virtues: A Handbook and Classification.* Washington, DC: American Psychological Association.

[B119] Pfeifer J. Moore W. Oswald T. Masten C. Mazziotta J. Iacoboni M. (2011). Entering adolescence: resistance to peer influence, risky behavior, and neural changes in emotion reactivity. *Neuron* 69 1029–1036. 10.1016/j.neuron.2011.02.019 21382560 PMC3840168

[B120] Plant K. M. Sanders M. R. (2007). Predictors of care-giver stress in families of preschool-aged children with developmental disabilities. *J. Intellect. Disabil. Res.* 51 109–124. 10.1111/j.1365-2788.2006.00829.x 17217475

[B121] Polanczyk G. V. Salum G. A. Sugaya L. S. Caye A. Rohde L. A. (2015). Annual research review: a meta-analysis of the worldwide prevalence of mental disorders in children and adolescents. *J. Child Psychol. Psychiatry.* 56 345–365. 10.1111/jcpp.12381 25649325

[B122] Proctor C. Tsukayama E. Wood A. M. Maltby J. Eades J. F. Linley P. A. (2011). Strengths gym: the impact of a character strengths-based intervention on the life satisfaction and wellbeing of adolescents. *J. Posit. Psychol.* 6 377–388. 10.1080/17439760.2011.594079

[B123] Rath T. (2007). *StrengthsFinder 2.0.* New York, NY: Gallup Press.

[B124] Raudenbush S. W. (2001). Comparing personal trajectories and drawing causal inferences from longitudinal data. *Annu. Rev. Psychol.* 52 501–525. 10.1146/annurev.psych.52.1.501 11148315

[B125] Rimehaug T. Wallander J. Berg-Nielsen T. S. (2011). Group and individual stability of three parenting dimensions. *Child Adolesc. Psychiatry Men. Healt* 5:19. 10.1186/1753-2000-5-19 21609442 PMC3125323

[B126] Robin A. L. Foster S. L. (2002). *Negotiating Parent-Adolescent Conflict: A Behavioral-Family Approach*. New York, NY: Guilford Press.

[B127] Rodríguez S. A. Perez-Brena N. J. Updegraff K. A. Umaña-Taylor A. J. (2014). Emotional closeness in mexican-origin adolescents’ relationships with mothers, fathers, and same-sex friends. *J. Youth Adolescenc* 43 1953–1968. 10.1007/s10964-013-0004-8 23999997 PMC4053492

[B128] Rusk R. Waters L. (2013). Tracing the size, reach, impact and breadth of positive psychology. *J. Posit. Psychol.* 8 207–221. 10.1080/17439760.2013.777766

[B129] Rusk R. D. Waters L. (2015). A psycho-social system approach to well-being: empirically deriving the five domains of positive functioning. *J. Posit. Psychol.* 10 141–152. 10.1080/17439760.2014.920409

[B130] Rutter M. Smith D. J. (1995). *Psychosocial Disorders in Young People?: Time Trends and Their Causes*. New York, NY: J. Wiley.

[B131] Sağkal A. Özdemir Y. (2019). Strength-based parenting and adolescents’ psychological outcomes: the role of mental toughness. *J. Psychol. Couns. Sch.* 1–13. 10.1017/jgc.2019.2

[B132] Sanders M. R. Markie-Dadds C. Tully L. A. Bor W. (2000). The triple P-positive parenting program: a comparison of enhanced, standard, and self-directed behavioral family intervention for parents of children with early onset conduct problems. *J. Consult. Clin. Psychol.* 68 624–640. 10.1037//0022-006x.68.4.624 10965638

[B133] Schnuck J. Handal P. J. (2011). Adjustment of college freshmen as predicted by both perceived parenting style and the five factor model of personality—personality and adjustment. *Psychology* 2 275–282. 10.4236/psych.2011.24044

[B134] Schutte N. S. Malouff J. M. (2018). The impact of signature character strengths interventions: a meta-analysis. *J. Happiness Stud.* 20 1179–1196 10.1007/s10902-018-9990-2

[B135] Schwartz O. S. Dudgeon P. Sheeber L. B. Yap M. B. Simmons J. G. Allen N. B. (2011). Observed maternal responses to adolescent behavior predict the onset of major depression. *Behav. Res. Ther.* 49 331–338. 10.1016/j.brat.2011.02.008 21440243

[B136] Schwartz O. S. Sheeber L. B. Dudgeon P. Allen N. B. (2012). Emotion socialization within the family environment and adolescent depression. *Clin. Psychol. Rev.* 32 447–453. 10.1016/j.cpr.2012.05.002 22717335

[B137] Seligman M. E. P. (1999). The APA 1998 annual report. *Am. Psychol.* 8 539–567.

[B138] Seligman M. E. P. Csikszentmihalyi M. (2000). Positive psychology: an introduction. *Am. Psychol.* 55 5–14. 11392865 10.1037//0003-066x.55.1.5

[B139] Shapiro A. (2004). The theme of the family in contemporary society and positive family psychology. *J. Fam. Psychother.* 15 19–38. 10.1016/j.midw.2018.02.022 29574301

[B140] Sheridan S. M. Warnes E. D. Cowan R. J. Schemm A. V. Clarke B. L. (2004). Family centered positive psychology: focusing on strengths to build student success. *Psychology in Schools* 41 7–17. 10.1002/pits.10134

[B141] Sheridan S. M. Burt J. D. (2009). “Family-centered positive psychology,” in *The Oxford Handbook of Positive Psychology*, 2nd Edn eds Lopez S. Snyder C. R. (Oxford: Oxford University).

[B142] Shortt A. L. Hutchinson D. M. Chapman R. Toumbourou J W. (2007). Family, school, peer and individual influences on early adolescent alcohol use: first-year impact of the resilient families programme. *Drug Alcohol Rev.* 26 625–634. 10.1080/09595230701613817 17943523

[B143] Singh K. (1998). Part-time employment in high school and its effect on academic achievement. *J. Educ. Res.* 91:131 10.1080/00220679809597533

[B144] Sisk C. L. Foster D. L. (2004). The neural basis of puberty and adolescence. *Nat. Neurosci.* 7 1040–1047. 10.1038/nn1326 15452575

[B145] Slade T. Johnston A. Oakley Brown M. A. Andrews G. Whiteford H. (2009). 2007 National survey of mental health and wellbeing: methods and key findings. *Aus. New Zealand J. Psychiatry* 43 594–605 10.1080/00048670902970882 19530016

[B146] Smollar J. Youniss J. (1985). Parent-adolescent relations in adolescents whose parents are divorced. *J. Early Adolesc.* 5 129–144. 10.1177/0272431685051011

[B147] Sodha S. (2009). *Kidulthood: life as a pre-teen in the UK today.* Avaliable at: https://www.actionforchildren.org.uk/media/3291/kidulthood_life_as_a_pre-teen_in_the_uk_today.pdf (accessed March 31, 2019).

[B148] Sodha S. Margo J. (2008). *Thursday’s Child.* London: Institute for Public Policy Research.

[B149] Somerville L. H. (2013). The teenage brain: sensitivity to social evaluation. *Curr. Dir. Psychol. Sci.* 22 121–127. 10.1177/0963721413476512 24761055 PMC3992953

[B150] Sowell E. R. Thompson P. M. Tessner K. D. Toga A. W. (2001). Mapping continued brain growth and gray matter density reduction in dorsal frontal cortex: inverse relationships during postadolescent brain maturation. *J. Neurosci.* 21 8819–8829. 10.1523/jneurosci.21-22-08819.2001 11698594 PMC6762261

[B151] Spreitzer G. Stephens J. P. Sweetman D. (2009). The reflected best self field experiment with adolescent leaders: exploring the psychological resources associated with feedback source and valence. *J. Posit. Psychol.* 4 331–348. 10.1080/17439760902992340

[B152] Stattin H. Klackenberg G. (1992). Discordant family relations in intact families: developmental tendencies over 18 years. *J. Marriage Fam.* 54 940–956. 10.2307/353174

[B153] Steinberg L. (1987). “Autonomy, conflict, and harmony in the family relationship,” in *At the threshold: The developing adolescent*, eds Feldman S. S. Elliot G. R. (Cambridge, MA: Harvard University Press), 255–276.

[B154] Steinberg L. (2000). We know some things: parent-adolescent relations in retrospect and prospect. *J. Res. Adolesc.* 11 1–19. 10.1111/1532-7795.00001

[B155] Steinberg L. (2001). We know some things: parent–adolescent relationships in retrospect and prospect. *J. Adolesc. Res.* 11 1–19. 10.1111/1532-7795.00001

[B156] Steinberg L. (2008). A social neuroscience perspective on adolescent risk-taking. *Dev. Rev.* 28 78–106. 10.1016/j.dr.2007.08.002 18509515 PMC2396566

[B157] Steinberg L. (2014). *The Age of Opportunity.* New York, NY: Houghton Mifflin Harcourt.

[B158] Steinberg L. Lamborn S. Dornbusch S. Darling N. (1992). Impact of parenting practices on adolescent achievement: authoritative parenting, school involvement, and encouragement to succeed. *Child Dev.* 63 1266–1281. 10.1111/j.1467-8624.1992.tb01694.x 1446552

[B159] Steinberg L. Lamborn S. D. Darling N. Mounts N. S. Dornbusch S. M. (1994). over-time changes in adjustment and competence among adolescents from authoritative, authoritarian, indulgent, and neglectful families. *Child Dev.* 65 754–770. 10.1111/j.1467-8624.1994.tb00781.x 8045165

[B160] Steinberg L. Morris A. S. (2001). Adolescent development. *Annu. Rev. Psychol.* 52 83–110. 10.1146/annurev.psych.52.1.83 11148300

[B161] Steinberg L. Silverberg S. B. (1986). The vicissitudes of autonomy in early adolescence. *Child Dev.* 57 841–851. 10.2307/1130361 3757604

[B162] Suldo S. M. Huebner E. S. (2004). ‘The role of life satisfaction in the relationship between authoritative parenting dimensions and adolescent problem behavior’. *Soc. Indic. Res.* 66 165–195. 10.1023/b:soci.0000007498.62080.1e

[B163] Suldo S. M. Savage J. A. Mercer S. H. (2014). Increasing middle school students’ life satisfaction: efficacy of a positive psychology group intervention. *J. Happiness Stud.* 15 19–42. 10.1007/s10902-013-9414-2

[B164] Sweeting H. Young R. West P. (2009). GHQ increases among scottish 15 year olds 1987-2006. *Soc. Psychiatry Psychiatric Epidemiol.* 44 579–586. 10.1007/s00127-008-0462-6 19037574 PMC2693777

[B165] Tabachnick B. G. Fidell L. S. Osterlind S. J. (2001). *Using Multivariate Statistics.* New York, NY: Pearsons.

[B166] Twenge J. M. Carter N. T. Campbell W. K. (2015). Time period, generational, and age differences in tolerance for controversial beliefs and lifestyles in the United States, 1972–2012. *Soc. Forces* 94 379–399. 10.1093/sf/sov050

[B167] Twenge J. M. Gentile B. DeWall N. C. Ma D. Lacefield K. Schurtz D. R. (2010). Birth cohort increases in psychopathology among young Americans, 1938–2007: a cross-temporal meta-analysis of the MMPI. *Clin. Psychol. Rev.* 30 145–154. 10.1016/j.cpr.2009.10.005 19945203

[B168] Twenge J. M. Joiner T. E. Rogers M. L. Martin G. N. (2018). Increases in depressive symptoms, suicide-related outcomes, and suicide rates among U.S. adolescents after 2010 and links to increased new media screen time. *Clin. Psychol. Sci.* 6 3 10.1177/2167702617723376

[B169] Ullman C. Tatar M. (2001). Psychological adjustment among Israeli adolescent immigrants: a report on life satisfaction, self-concept, and self-esteem. *J. Youth Adolesc.* 30 449–463. 10.1023/a:1010445200081

[B170] Vaillant G. Mukamal K. (2001). Successful aging. *Am. J. Psychiatry* 158 839–847. 11384887 10.1176/appi.ajp.158.6.839

[B171] Vazire S. (2010). Who knows what about a person? The self-other knowledge asymmetry (SOKA) model. *J. Personal. Soc. Psychol.* 98 281–300. 10.1037/a0017908 20085401

[B172] Vazire S. Mehl M. R. (2008). Knowing me, knowing you: the accuracy and unique predictive validity of self-ratings and other-ratings of daily behavior. *J. Personal. Soc. Psychol.* 95 1202–1216. 10.1037/a0013314 18954202

[B173] Viner R. M. Taylor B. (2007). Adult outcomes of binge drinking in adolescence: findings from a UK national birth cohort. *J. Epidemiol. Commun. Health* 61 902–907. 10.1136/jech.2005.038117 17873228 PMC2652971

[B174] Walkner A. J. Rueter M. A. (2014). Adoption status and family relationships during the transition to young adulthood. *J. Fam. Psychol.* 28 877–886. 10.1037/fam0000020 25221972 PMC4307793

[B175] Waters L. (2015a). Strength-based parenting and life satisfaction in teenagers. *Adv. Soc. Sci. Res. J.* 2 158–173. 10.14738/assrj.211.1651

[B176] Waters L. (2015b). The relationship between strength-based parenting with children’s stress levels and strength-based coping approaches. *Psycholog* 6 689–699. 10.4236/psych.2015.66067

[B177] Waters L. (2016). The relationship between child stress, child mindfulness and parent mindfulness. *Psycholog* 7 40–51. 10.4236/psych.2016.71006

[B178] Waters L. (2017). *The Strengths Switch.* North Sydney, NSW: Ebury Press.

[B179] Waters L. (in press). Using positive psychology interventions to strengthen family happiness: a family systems approach. *J. Posit. Psychol.*

[B180] Waters L. Sun J. (2016). Can a brief strength-based parenting intervention boost self-efficacy and positive emotions in parents? *Int. J. Appl. Posit. Psychol.* 1 41–56. 10.1007/s41042-017-0007-x

[B181] Waters L. E. Loton D. Jack H. K. (2019). Does strength-based parenting predict academic achievement? The mediating effects of perseverance and engagement. *J. Happiness Stud.* 20 1121–1140. 10.1007/s10902-018-9983-1

[B182] Whittle S. Simmons J. G. Dennison M. Vijayakumar N. Schwartz O. Allen N. B. (2014). Positive parenting predicts the development of adolescent brain structure: a longitudinal study. *Dev. Cognit. Neurosci.* 8 7–17. 10.1016/j.dcn.2013.10.006 24269113 PMC6990097

[B183] Willinger U. Diendorfer-Radner G. Willnauer R. Jörgl G. Hager V. (2005). Parenting stress and parental bonding. *Behav. Med.* 31 63–72. 10.3200/bmed.31.2.63-72 16130308

[B184] World Health Organization (2014). *Health for the World’s Adolescents.* Geneva: WHO.

[B185] Yap M. B. H. Whittle S. Yücel M. Sheeber L. B. Pantelis C. Simmons J. G. (2008). Parenting experiences interact with brain structure to predict depressive symptoms in adolescents. *Arch. Gen. Psychiatry* 65 1377–1385. 19047524 10.1001/archpsyc.65.12.1377

